# One‐Pot Transition‐Metal‐Free Methods for the Synthesis of All Three Bonds of the Alkyne's Triple Bond

**DOI:** 10.1002/chem.202503062

**Published:** 2026-01-10

**Authors:** Eugene Kim, Yu Wu, Judith N. Currano, Jianyou Mao, Patrick J. Walsh

**Affiliations:** ^1^ Department of Chemistry University of Pennsylvania 213 S. 34th St. Philadelphia PA 10104 USA; ^2^ School of Chemistry and Molecular Engineering Nanjing Tech University 30 South Puzhu Road Nanjing 211816 P. R. China

**Keywords:** acetylenes, internal alkynes, one‐pot syntheses, Sonogashira cross‐coupling, terminal alkynes

## Abstract

Alkynes are among the most useful functional groups in synthetic chemistry, as they are excellent starting materials for various reactions and serve as valuable precursors during total synthesis. Despite the high number of transformations that utilize alkynes as substrates, few methods actually exist to synthesize them. Known methods involve several synthetic steps, harsh conditions, or cryogenic temperatures, which render them inconvenient and inefficient. This review of alkyne syntheses cover methods that enable the formation of all three bonds of the alkyne's triple bond in a transition‐metal‐free, one‐pot fashion. This review offers a guide to gauge if current methods are potentially suitable to be scaled and used in industry and pharmaceutical processes.

## Graphical abstract

One‐pot transition‐metal‐free methods for the synthesis of all three bonds of the alkyne's triple bond are reviewed. After analysis of several named reactions and discussion of improvements, recent advances in the synthesis of alkynes are discussed. Each section contains a proposed reaction mechanism and analysis of the strengths and weaknesses of the approaches.

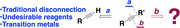

## 1 Introduction

**Scheme 1:**
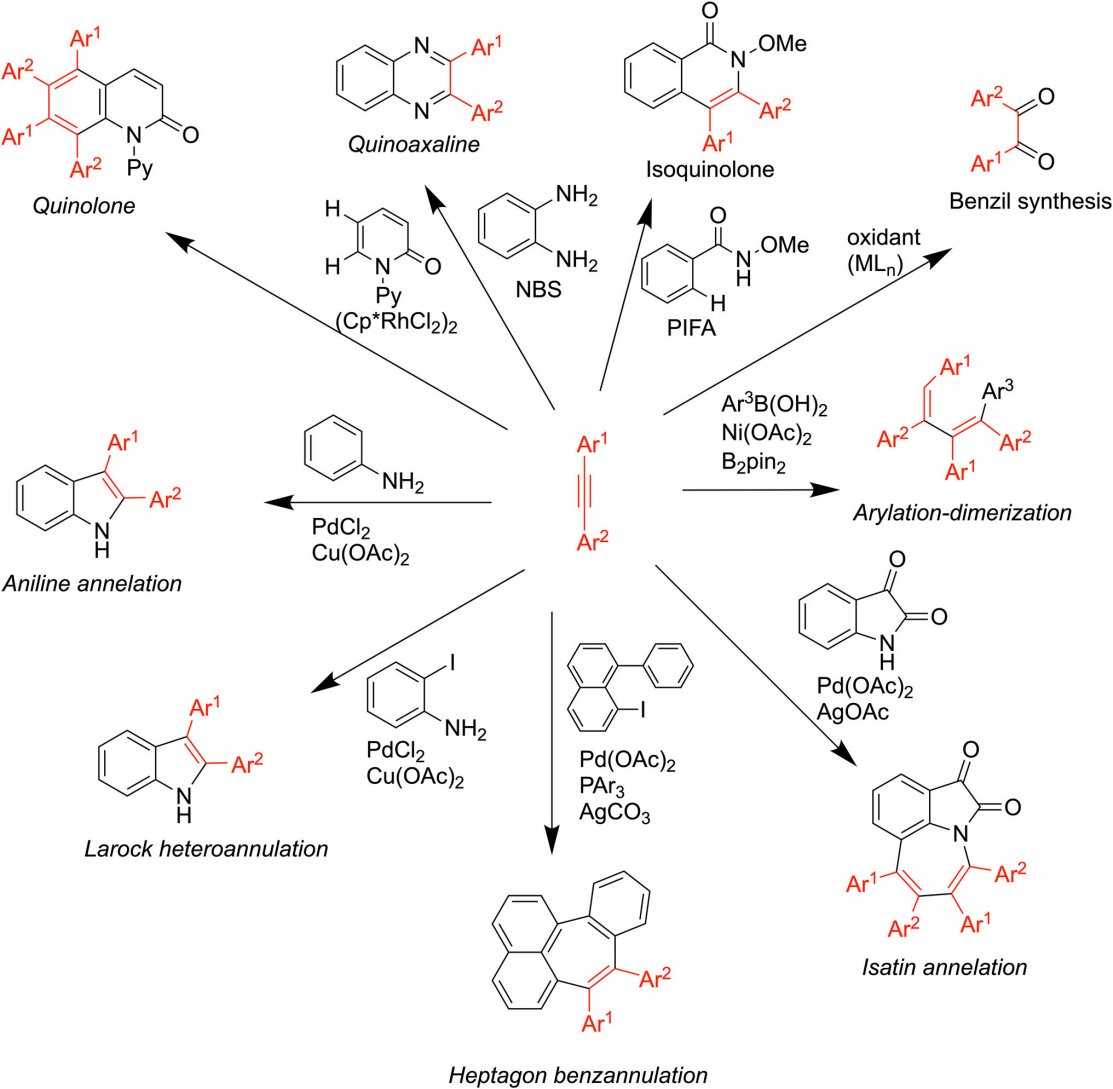
Alkyne transformations.

**Figure 1:**
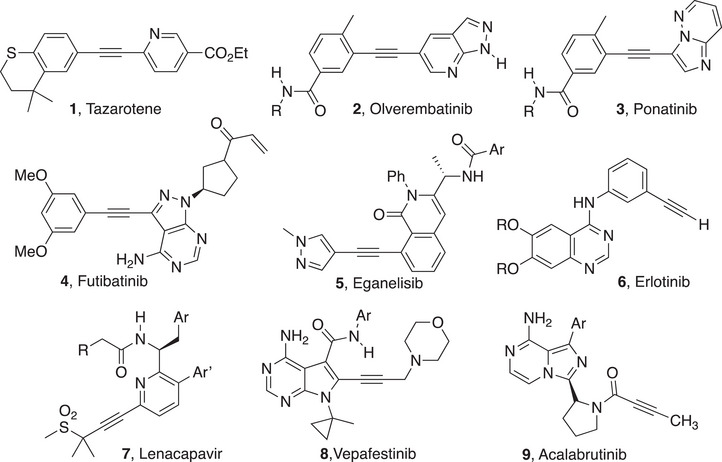
Alkyne‐based medications.

Alkynes have an array of uses in synthetic chemistry, often acting as a building block for larger compounds. This is due to alkynes being highly reactive, making them one of the most useful functional groups. Their high reactivity stems from the low bond strength of the second π‐bond and the lower energy of the π* orbital, making scores of reactions with the alkyne π‐system sufficiently exergonic to drive bond‐forming processes. Alkynes make excellent starting materials for many cycloaddition reactions such as click chemistry,^[^ ^1^ ^]^ transition‐metal‐catalyzed processes,^[^ ^2^, ^3^, ^4^, ^5^ ^]^ multicomponent reactions,^[^ ^6^ ^]^ and radical cascade reactions (Scheme 1).^[^ ^7^ ^]^ They can even behave as carbonyl equivalents.^[^ ^8^ ^]^ In addition to their synthetic utility and versatility, alkynes are prevalent in biological^[^ ^9^ ^]^ and medicinal^[^ ^10^ ^]^ compounds, as well as natural products.^[^ ^11^, ^12^ ^]^ Several biologically active agents and medications that contain carbon‐carbon triple bonds^[^ ^13^ ^]^ are shown in Figure 1.

Due to the increasing relevancy of alkynes, there is a high demand for more efficient methods for their synthesis. Existing methods present issues of sustainability, as they employ transition metal complexes that are often based on noble metals. This reliance on scarce materials^[^ ^14^ ^]^ will prove problematic in the long run, especially in industry‐scale productions. Newer metal‐free syntheses of alkynes will surpass those that use transition‐metal catalysts, which are becoming more expensive.

Herein, we present a review on the synthesis of alkynes. Given the importance of this reactive functional group, their synthesis has been previously reviewed. Past authors have focused on alkynes from nonalkyne sources,^[^ ^15^ ^]^ alkynes originating from carbonyls,^[^ ^16^ ^]^ and the Sonogashira coupling.^[^ ^17^ ^]^ However, no review has centered on methods of directly synthesizing the alkyne. The focus of this review is to explore the *one‐pot, metal‐free synthesis of alkynes that form all three bonds of the alkyne*. To qualify, a publication will have to show at least three examples. We will present several methods along with discussions on their strengths and potential areas for further improvement to allow for assessment of their sustainability and suitability for both laboratory and industry‐scale usage. As such, this review will not cover transition‐metal‐catalyzed procedures such as the venerable Sonogashira coupling to functionalize alkynes^[^ ^17^ ^]^ or alkyne metathesis.^[^ ^18^, ^19^ ^]^ Although double elimination strategies comprise useful methods to generate triple bonds, approaches such as alkene bromination and double elimination to the alkyne will be excluded because only two of the three bonds of the triple bond are formed. This article will first briefly analyze the literature of alkyne syntheses by year, documenting the total number of alkyne syntheses. Next, commonly used early developments in alkyne syntheses, like the Corey‐Fuchs and Bestmann‐Ohira reactions, will be discussed before describing more recent one‐pot syntheses of alkynes. In this context, the synthesis of terminal alkynes will precede the description of the preparation of internal alkynes.

## 2 Analysis of the Literature

**Figure 2:**
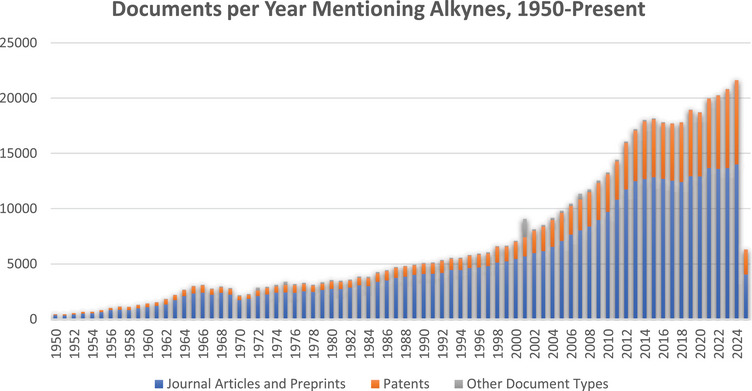
Number of documents per year in which Chemical Abstracts Service indexed an alkyne. Documents are divided into three categories: journal articles and preprints, patents, and other document types.

To gauge the scope of the literature surrounding alkynes and their synthesis, a series of searches were run in Chemical Abstracts Service's SciFinder product between April and June, 2025. A full description of the search strategies and their results appears in the Supporting Information. From 1831 to 2025, a total of 3.35 million alkyne‐containing substances were indexed in a total of 543,198 references. Although such substances appear in the literature dating back to the 1830s, the number of documents per year mentioning alkyne‐containing substances began to increase steadily around 1950, with a steeper climb beginning in the early 2000s (Figure 2).

These substances show a wide variety of uses, but only 212,890 are currently commercially available (data from CHEMCATS, one of the databases searched by SciFinder). Of the remaining substances, a total of 566,983 are neither commercially available nor have a published synthesis indexed by CAS, indicating that there are many new targets in need of effective synthetic methods.

### 2.1 Syntheses of Alkynes

As this review focuses on methods of forming all three bonds of the alkyne without the use of transition metal catalysis, a search of the literature for past practices in such syntheses was next undertaken. These searches were also performed in SciFinder and were broken into two categories: synthesis of terminal alkynes (H─C≡C─R, where R≠H) and synthesis of “internal” alkynes (R^1^─C≡C─R^2^, where R^1^, R^2^ ≠ H). For comparison purposes, we also include Sonogashira‐type reactions, because it is one of the most common reactions to make arylated internal alkynes. Details of all searches appear in the Supporting Information.

**Figure 3:**
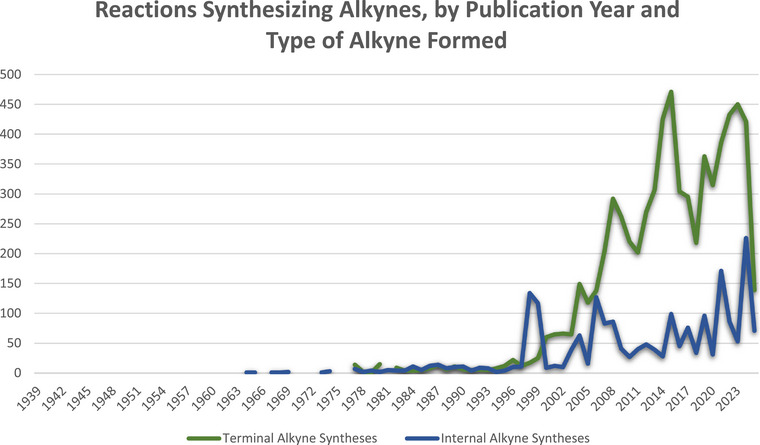
Number of SciFinder‐indexed reactions per year in which a substance containing a terminal alkyne (RC≡CH) or an internal alkyne (RC≡CR) is prepared through the construction of all three bonds of the alkyne. Baseline gaps appear where no reactions were published in that year.

The synthesis of terminal alkynes was first examined, which consisted of a search for the formation of all three bonds between the two carbons in a single step, with some reactions where multiple steps were taken but no intermediates isolated. The searches retrieved 6855 reactions. As noted earlier, metallic elements were excluded from the product, as were reactions that employed catalysts containing transition metals, lanthanides, and actinides. These reactions appeared in 3170 publications, spanning the years 1955–2025, with a huge jump in the number of published reactions occurring in 2004 (Figure 3). The second set of searches retrieved 2067 reactions that synthesized internal alkynes through the formation of all three bonds in a single step, although some reactions also included multiple stages. Once again, no metallic elements were permitted in the product, and reactions employing transition metal, lanthanide, and actinide catalysts were excluded. These reactions appeared in 460 references, spanning the years 1938–2025, which climbed steadily, with occasional spikes and dips (Figure 3).

### 2.2 Methods to Synthesize Terminal Alkynes

Starting with the set of 6855 reactions for the synthesis of terminal alkynes, we next examined general conditions under which they were made. For the most part, the reactions ran in moderate to high yields; most of the reactions for which a yield was indexed in SciFinder had yields over 50% (84%), and a good number had yields above 80% (39% of those indexed).

The largest subset of the results (5312 reactions) employed reactants with the (MeO)_2_P(═O)─C substructure. Furthermore, the vast majority of these results (4809) convert an aldehyde to the terminal alkyne using potassium carbonate or cesium carbonate, often in methanol, and in high yield (43% of the retrieved reactions reported a yield of 80% or higher). This result could be interpreted as arising from reactions like the Bestmann‐Ohira. Reactions that do not use this class of phosphorus reactant tend to employ butyllithium and triphenylphosphine, which are key components of the Corey‐Fuchs approach.

### 2.3 Methods to Prepare Internal Alkynes

We next examined the set of 2067 reactions that synthesized the internal alkynes. Of these reactions 1500 had an indexed yield, and, of this subset, 84% of the reactions had yields over 50%, with 39% of the reactions having yields above 80%. Extracting trends in reaction conditions was challenging due to the functionality of the database, but it was possible to determine that the most used reagent was triphenylphosphine. Of the 618 reactions, that employed triphenylphosphine, the majority also used butyllithium (61%). These results point to the Corey‐Fuchs reaction, most likely followed by alkylation of the in situ formed acetylide. These reactions will be outlined below.

### 2.4 Comparison to Sonogashira‐Type Reactions

**Figure 4:**
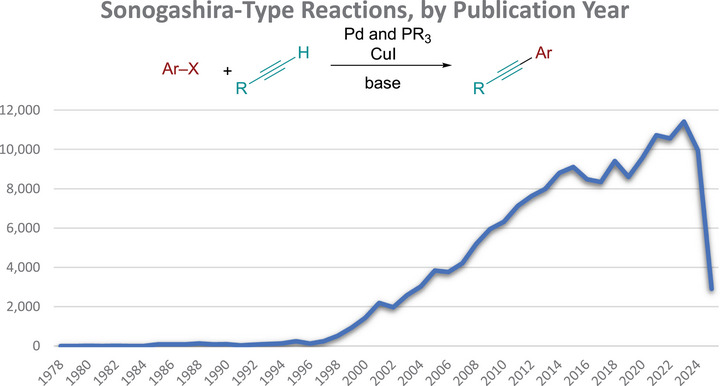
Number of SciFinder‐indexed reactions per year that employ a Sonogashira‐type coupling. Baseline gaps appear where no reactions were published in that year.

As a point of comparison, we explored the number of Sonogashira‐type reactions in the SciFinder reaction database. The total number of reactions, 174,174, was much larger than either the set of the terminal alkyne syntheses or the set of internal alkyne syntheses without the use of transition‐metal catalysts. However, the graph of Sonogashira‐type reactions published per year shows publication trends that are similar to the publication of reactions that form RC≡CH and RC≡CR substances without the use of metal catalysts (Figure 4). This result suggests that alkynes containing at least one aryl group are typically prepared by palladium and copper co‐catalyzed Sonogashira reactions. The utility and reliability of this reaction, however, are counterbalanced by the use of the precious metal palladium, the toxicity of copper, the use of phosphine ligands, and the need to purchase or prepare the terminal alkyne. Here, alternative one‐pot methods to make all three bonds of arylated internal alkynes stand to have the greatest impact.

## 3 Named Reactions

Named reactions will be reviewed here for the purpose of comparison with more recent advances. Reaction mechanisms will also be discussed.

### 3.1 Corey‐Fuchs Reaction

**Scheme 2:**
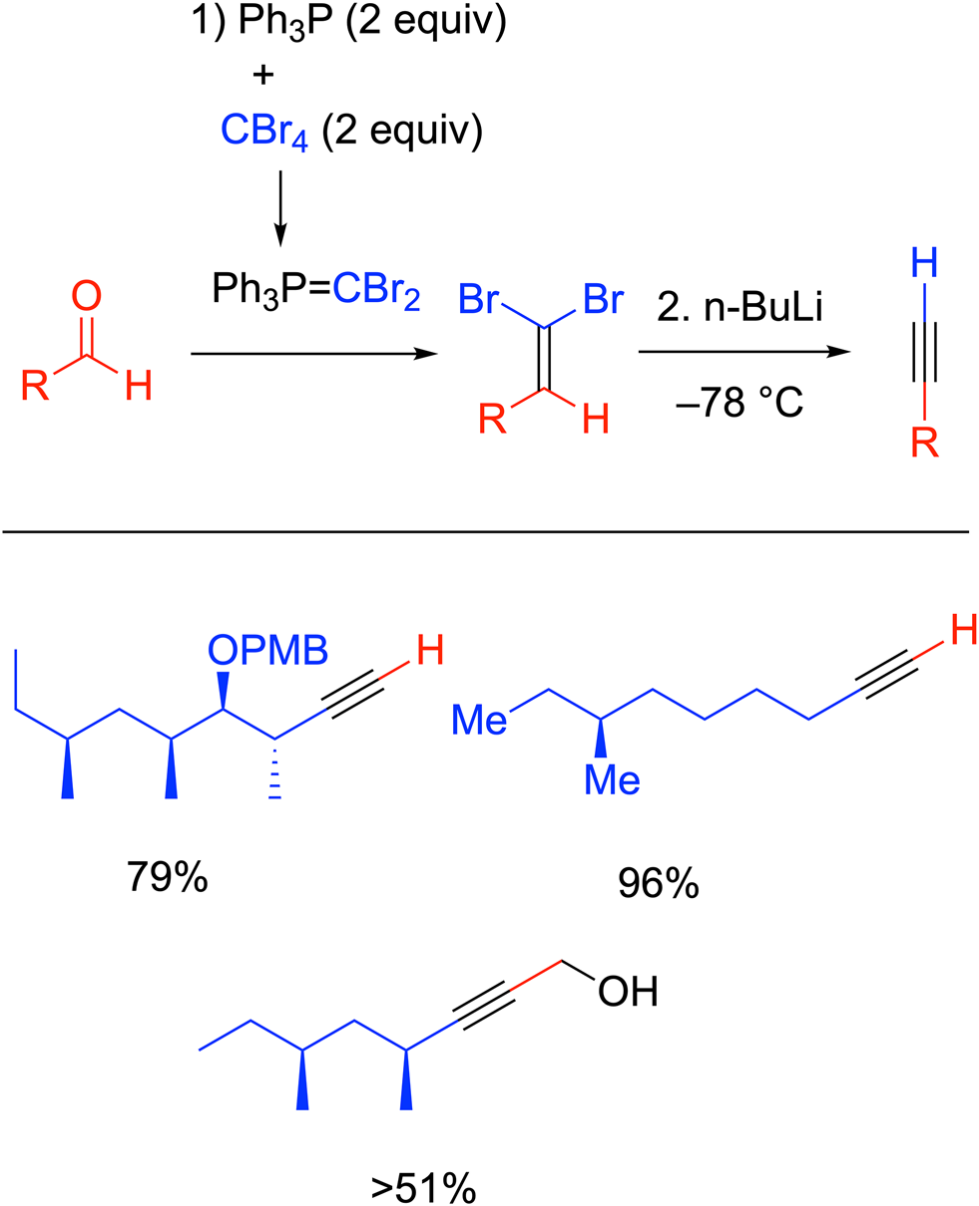
Corey‐Fuchs reaction and selected examples^[^ ^24^, ^25^ ^]^

The Corey‐Fuchs reaction^[^ ^20^ ^]^ to synthesize terminal alkynes was historically significant because it was the first useful procedure for forming alkynes from aldehydes with good yields (Scheme 2).^[^ ^21^ ^]^ This synthesis begins with a reaction between 2 equiv each of triphenylphosphine and CBr_4_ for the in situ formation of an equivalent of the dibromo ylide, Ph_3_P═CBr_2_. The ylide is used to perform a Wittig reaction with the aldehyde to form a dibromoalkene intermediate. This first step is known as the Ramirez synthesis of 1,1‐dibromo alkenes.^[^ ^22^ ^]^ Next, the dibromo alkene is treated with *n*‐butyllithium at low temperature (often −78 °C) to form the alkyne.^[^ ^20^ ^]^ In some cases, this two‐step procedure is performed with isolation of the 1,1‐dibromo alkene, but in others it can be carried out in one pot. The Corey‐Fuchs reaction has been used extensively^[^ ^21^ ^]^ in total synthesis,^[^ ^23^ ^]^ and some representative examples are shown in Scheme 2, including the synthesis of alkynes as precursors toward natural products like metacridamide B^[^ ^24^ ^]^ and (+)‐violapyrone C.^[^ ^25^ ^]^ An important feature of the Corey‐Fuchs reaction is that the product is an acetylide that can be quenched with different electrophiles to extend the carbon framework to give internal alkynes. An example of such a process is shown in the synthesis of putative orevactaene,^[^ ^26^ ^]^ where the 51% yield was recorded over three steps (oxidation to the aldehyde, Corey‐Fuchs, and reaction with (CH_2_O)_n_).

**Scheme 3:**
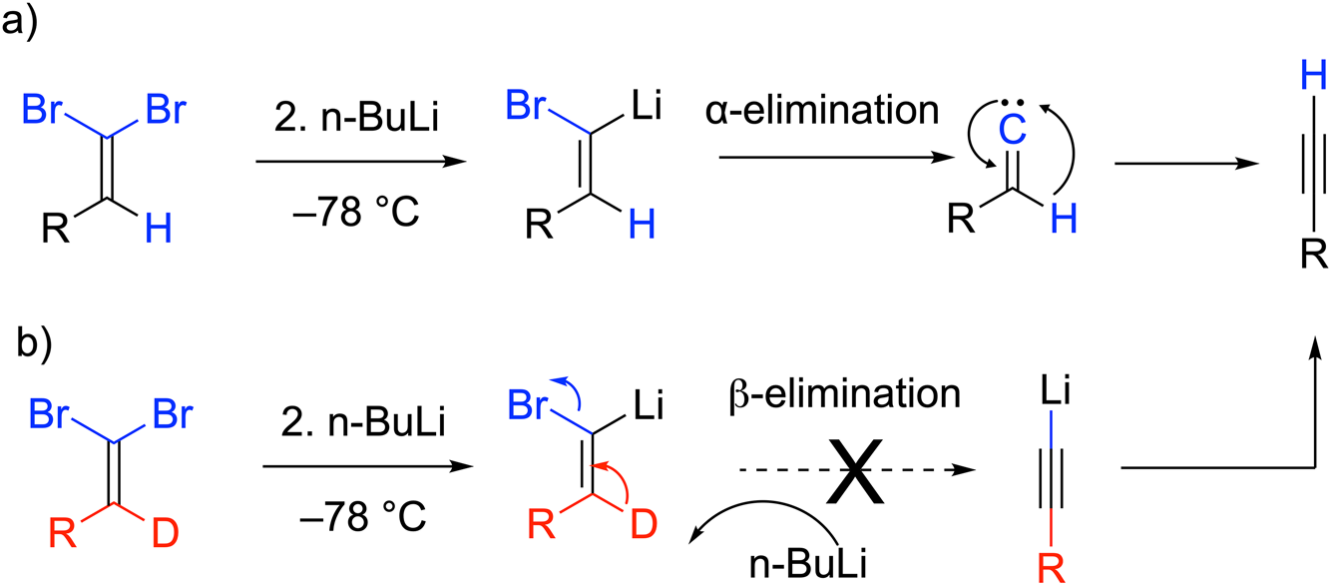
Key steps in the mechanism of the Corey‐Fuchs reaction.

Mechanistically, the first step of the Corey‐Fuchs reaction is a Wittig reaction of the dibromo ylide with the aldehyde to form an oxaphosphetane, which then reacts to form the dibromoalkene. Mechanistic studies performed by Namboothiri and coworkers investigated the action of *n*‐butyllithium on a deuterium‐labeled 1,1‐dibromo alkene substrate (RCD═CBr_2_) compared to the protio analog.^[^ ^27^ ^]^ A lithium‐halogen exchange occurs between the 1,1‐dibromo alkene and the *n*‐BuLi, followed by an α‐elimination to form a vinylidene (Scheme 3a). This carbene then isomerizes via a 1,2 shift (Fritsch‐Buttenberg‐Wiechell rearrangement) to form the alkyne product. A mechanism where the metalated alkene (RCD═CBrLi) underwent reaction with another *n*‐BuLi in an E2 fashion to the acetylide would result in loss of the deuterium label (Scheme 3b) and is inconsistent with the observation of the deuterium label in the product.

Despite its popularity, the original Corey‐Fuchs reaction is not without its drawbacks. The reaction is not atom economical, using a minimum of 2 equiv PPh_3_ and CBr_4_. The use of strong bases, such as *n*‐butyllithium, and cryogenic temperatures makes the Corey‐Fuchs synthesis inconvenient to scale up. These issues have inspired improvements to the original procedure, some of which are highlighted below.

#### 3.1.1 Modified Corey‐Fuchs Reactions

**Scheme 4:**
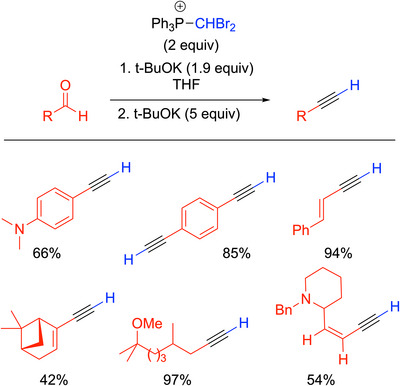
Synthesis of alkynes with dibromomethylphosphonium bromide.

As stated before, the Corey‐Fuchs reaction requires two steps to form alkynes: a Ramirez‐like olefination to generate the dibromoalkene and elimination of the dibromoalkene to yield the alkyne. Michel, Rassat, and others sought to avoid the intermediate step of the Corey‐Fuchs reaction. Dibromomethylphosphonium bromide ([Ph_3_PCHBr_2_]^+^) was utilized to afford alkynes from aldehydes.^[^ ^28^ ^]^ Under an argon atmosphere, the aldehyde (1 equiv) was added to [Ph_3_PCHBr_2_]^+^ (2 equiv) and KO^t^Bu (1.9 equiv) in THF to generate a solution of the ylide, Ph_3_P═CBr_2_. After 10 minutes, additional KO^t^Bu (5 equiv) was added to the reaction mixture. The temperatures employed depended on the substrates, as some were conducted at −78 °C while others were run at room temperature (Scheme 4). The procedure outlined by Rassat and colleagues is an alternative to the Corey‐Fuchs that doesn't use the harsh reagents *n*‐BuLi or CBr_4_. The scope of the reaction, however, leaves room for improvement.

#### 3.1.2 DBU‐Mediated Corey‐Fuchs

**Scheme 5:**
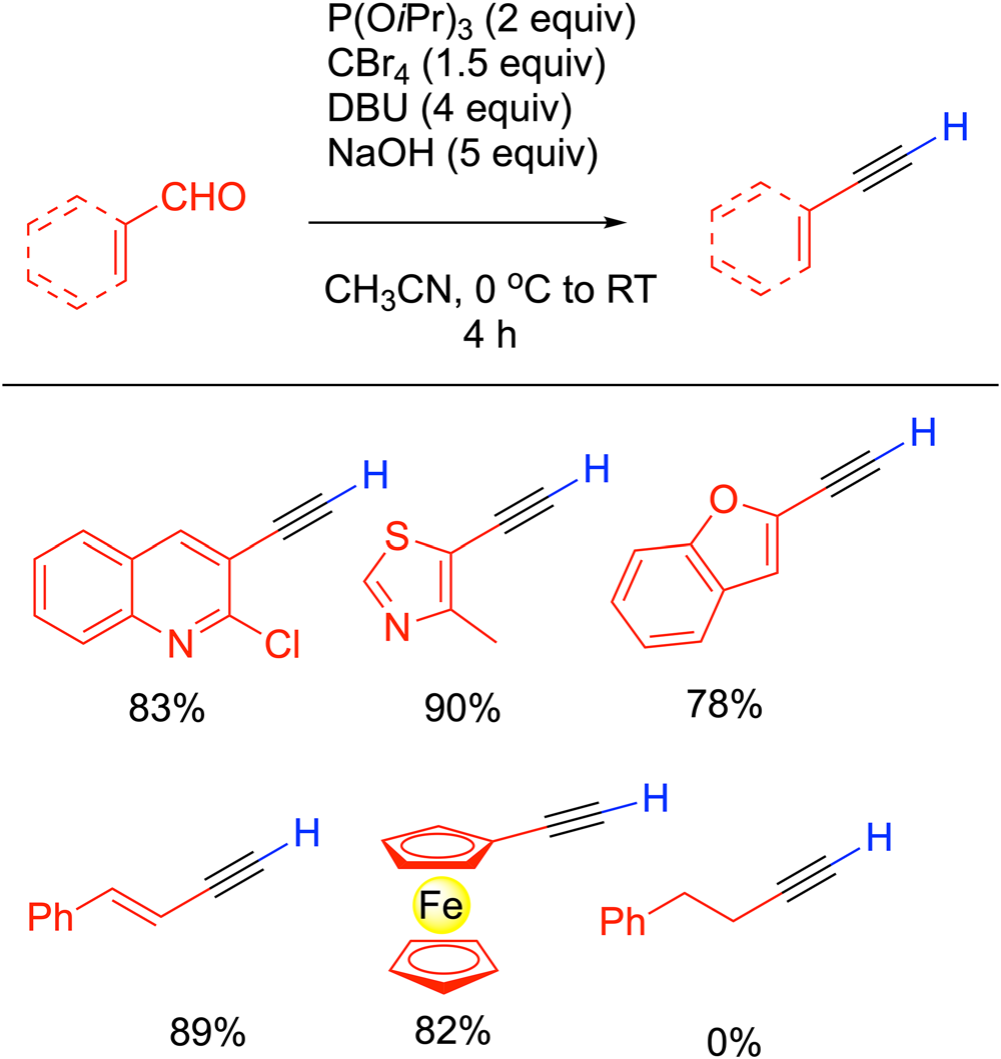
Doddi's modification of the Corey‐Fuchs reaction.

A noteworthy modification of the Corey‐Fuchs reaction was introduced by Doddi and coworkers,^[^ ^29^ ^]^ who sought to modify the two‐step process so that it could be conducted in one pot under milder conditions (Scheme 5). This was done by employing Lauten's^[^ ^30^ ^]^ modified Ramirez olefination^[^ ^22^ ^]^ using CBr_4_ and P(O*^i^* Pr)_3_ (instead of PPh_3_). Additionally, elimination conditions with DBU and NaOH were substituted for the use of *n*‐BuLi in the original Corey‐Fuchs procedure. The group found that the NaOH was the key because it was able to quench the electrophilic side product bromotriisopropoxyphosphonium bromide, Br_2_P(O*^i^* Pr)_3_, thereby raising the overall yield. NaOH also sped up the rate of the reaction, allowing complete conversion in 4 hours. The DBU is proposed to be responsible for both the elimination to give the 1‐bromo alkyne and its debromination via nucleophilic attack on the Br of the C─Br bond.^[^ ^31^ ^]^

The procedure was found to tolerate electron‐donating and electron‐withdrawing groups on benzaldehydes, providing terminal aryl acetylenes in high yields. Halogenated, polyaromatic, and heterocyclic aldehydes were synthesized in yields from 80–90% (for selected examples, see Scheme 5). Unfortunately, alkyl aldehydes were not viable substrates. The procedure introduced by Doddi^[^ ^29^ ^]^ has good yields and a wide substrate scope. It avoids some of the harsher reagents used in the original Corey‐Fuchs reaction. The convenience of the one‐pot procedure with milder conditions is attractive for forming terminal alkynes. As will be outlined below in Section 3.8 on 1‐haloalkynes, the Doddi procedure in Scheme 5 can be modified to yield a variety of 1‐bromo alkynes in high yields.^[^ ^29^ ^]^

### 3.2 Seyferth‐Gilbert Homologation

**Scheme 6:**
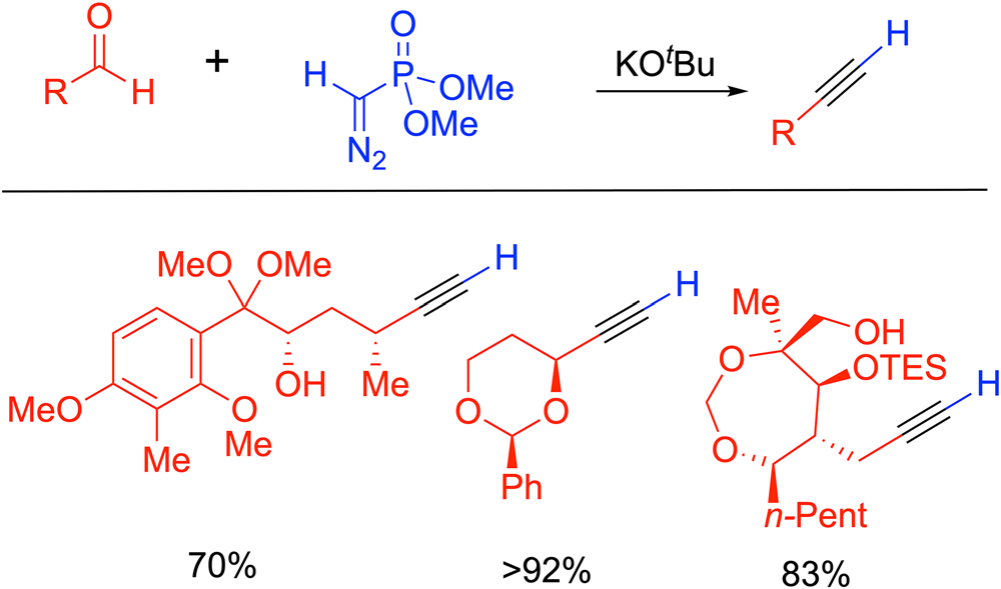
Seyferth‐Gilbert homologation and selected examples. Yields are over‐telescoped processes with two steps.^[^ ^37^, ^38^, ^39^ ^]^

In the quest for new and efficient methods for the synthesis of alkynes, Gilbert and Weerasooriya developed a general method for the one‐carbon homologation of aldehydes^[^ ^32^ ^]^ and ketones^[^ ^33^ ^]^ (Scheme 6). The reaction is based on the Seyferth reagent, dimethyl diazomethylphosphonate, (MeO)_2_P(═O)CHN_2_, which was originally applied to the synthesis of a few alkynes by Colvin^[^ ^34^ ^]^ and coworkers^[^ ^35^ ^]^ (see Section 3.5 for more details). This reaction has become known as the Seyferth‐Gilbert homologation.^[^ ^16^, ^36^ ^]^ It is performed by combining equimolar (MeO)_2_P(═O)CHN_2_ with the aldehyde or ketone and KO^t^Bu in THF at −78 °C for 12–16 hours then warming to room temperature to produce the alkyne. The Seyferth‐Gilbert reaction is tolerant of aldehydes and ketones bearing alpha‐C─H's and has been utilized in total syntheses to produce alkyne intermediates. Some examples (Scheme 6) include the synthesis of elisapterosin B, where a yield of 70% can be seen over the two‐step reaction. The first step is a DIBAL reduction of the lactone to the lactol, followed by subjection of this intermediate to the Seyferth‐Gilbert conditions.^[^ ^37^ ^]^ In an application of alkynes to the enantioselective synthesis of allenylsilanes, an aldehyde was converted to the enantioenriched terminal alkyne in > 92% (here, the yield was given over two steps: the Seyferth‐Gilbert and deprotonation of the product followed by trapping with TMS–Cl).^[^ ^38^ ^]^ Finally, studies on the total synthesis of formamicin,^[^ ^39^ ^]^ which shows an 83% yield over two steps: a Seyferth‐Gilbert reaction and a DIBAL reduction to remove a pivalate from the primary alcohol.

The mechanism of the Seyferth‐Gilbert reaction is very similar to that of the Bestmann‐Ohira, and as such will be discussed in further detail in Section 3.3.

While the Seyferth‐Gilbert reaction provides a new avenue for the synthesis of alkynes, it presents a few safety concerns with the use of the Seyferth reagent. The synthesis also has a total of five steps, including the use of tosyl azide, and utilizes cryogenic temperatures. These concerns inspired researchers to modify this procedure, bringing about new methods such as the Bestmann‐Ohira reaction.

### 3.3 Bestmann‐Ohira Reaction

**Scheme 7:**
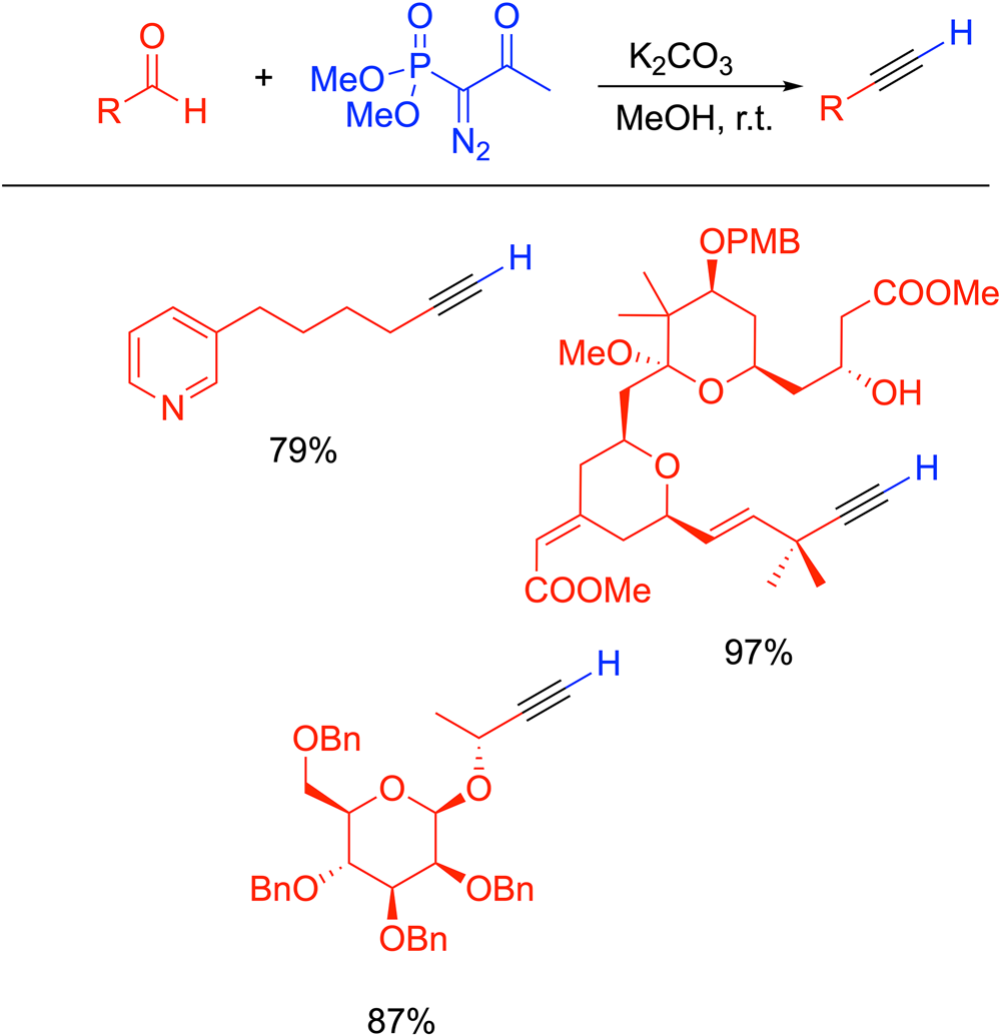
Bestmann‐Ohira reaction and selected examples.^[^ ^44^, ^45^, ^46^ ^]^

Building upon the Seyferth‐Gilbert reaction, Bestmann and Ohira developed a one‐pot method for the synthesis of alkynes from aldehydes under mildly basic conditions, now known as the Bestmann‐Ohira reaction (Scheme 7).^[^ ^40^ ^]^ Ohira found that the active anionic intermediate generated in the Seyferth‐Gilbert reaction could be accessed beginning with the more readily prepared (MeO)_2_P(═O)C(Ac)N_2_.^[^ ^41^ ^]^ Bestmann and coworkers^[^ ^42^, ^43^ ^]^ then explored the scope of the one‐carbon homologation of aldehydes to terminal alkynes. In the event, Ohira's phosphonate is added to an aldehyde solution containing K_2_CO_3_ in methanol at room temperature, resulting in cleavage of the acetyl group to form the nucleophilic intermediate in the Seyferth‐Gilbert reaction. The alkyne products are generally formed in high yields. The procedure was shown to tolerate various functional groups and does not disturb enantioenriched aldehyde substrates with α‐stereocenters.^[^ ^42^ ^]^ Selected examples are shown in Scheme 7, including applications in total synthesis for cytotoxic sponge alkaloids (79% yield),^[^ ^44^ ^]^ bryostatin 16 (97% yield),^[^ ^45^ ^]^ and colletotrichamide A (87% yield).^[^ ^46^ ^]^

**Scheme 8:**
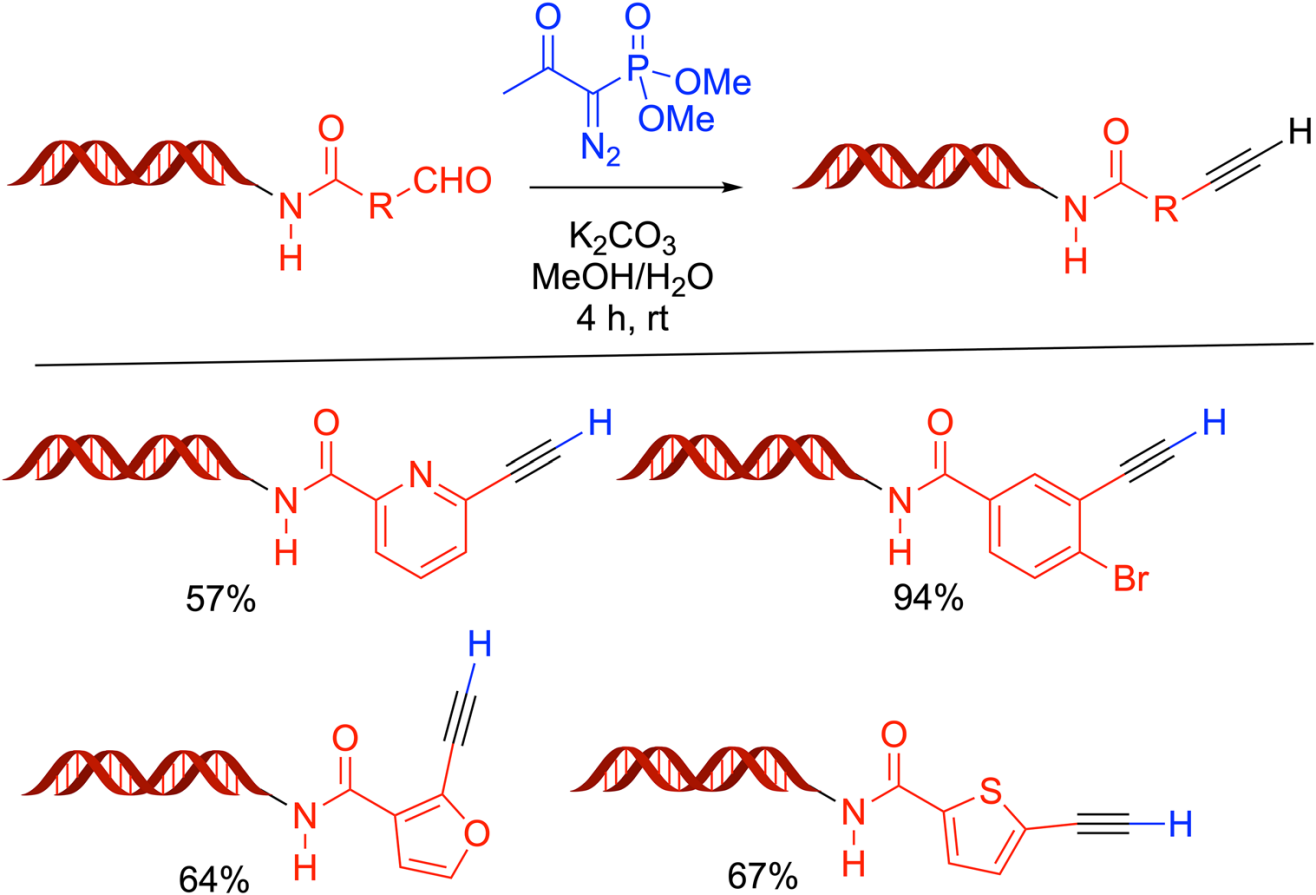
Application of the Bestmann‐Ohira reaction to DNA conjugated aldehydes.

An impressive recent application of the Bestmann‐Ohira reaction that demonstrated the versatility of this transformation involved its application on DNA (Scheme 8).^[^ ^47^ ^]^ An aldehyde conjugated to DNA reacted with the Bestmann‐Ohira reagent in a mixture of 80% methanol/20% water in the presence of K_2_CO_3_ for 4 hours at rt to furnish the desired terminal alkyne products in 57–94% yields.

**Scheme 9:**
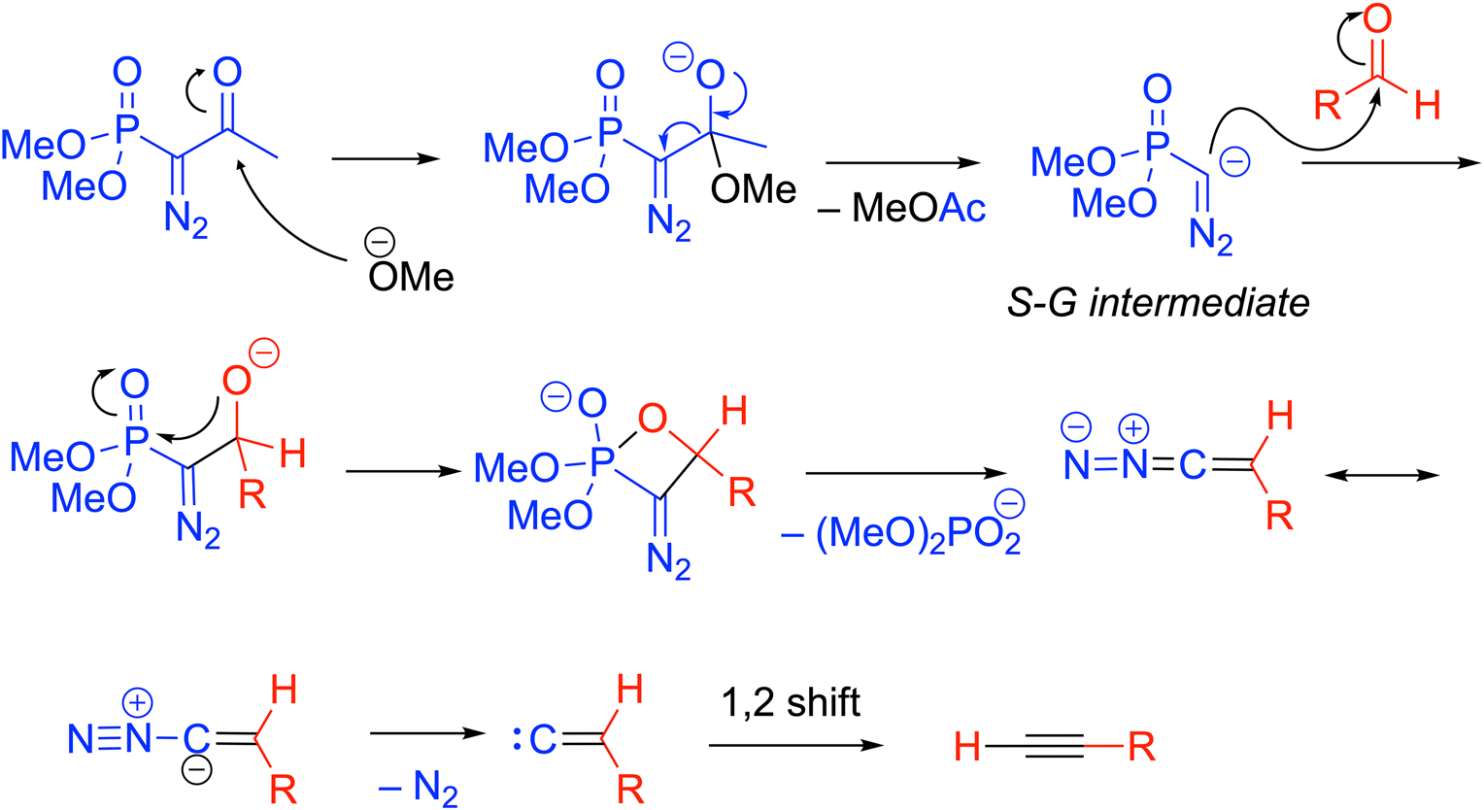
Proposed mechanism of the Bestmann‐Ohira reaction.

The mechanism of the Bestmann‐Ohira reaction (Scheme 9) follows similarly to that of the Seyferth‐Gilbert homologation,^[^ ^33^ ^]^ where methanol under basic conditions cleaves the acetyl group to generate the key intermediate in the Seyferth‐Gilbert reaction. Addition of this carbanion to the aldehyde, oxophosphetane formation, and loss of (MeO)_2_PO_2_^−^ forms the diazoalkene. The diazo intermediate then loses dinitrogen to generate a vinylidene (as seen in the Corey‐Fuchs reaction in Scheme 3) that rearranges to the alkyne via a 1,2‐H shift.

**Scheme 10:**
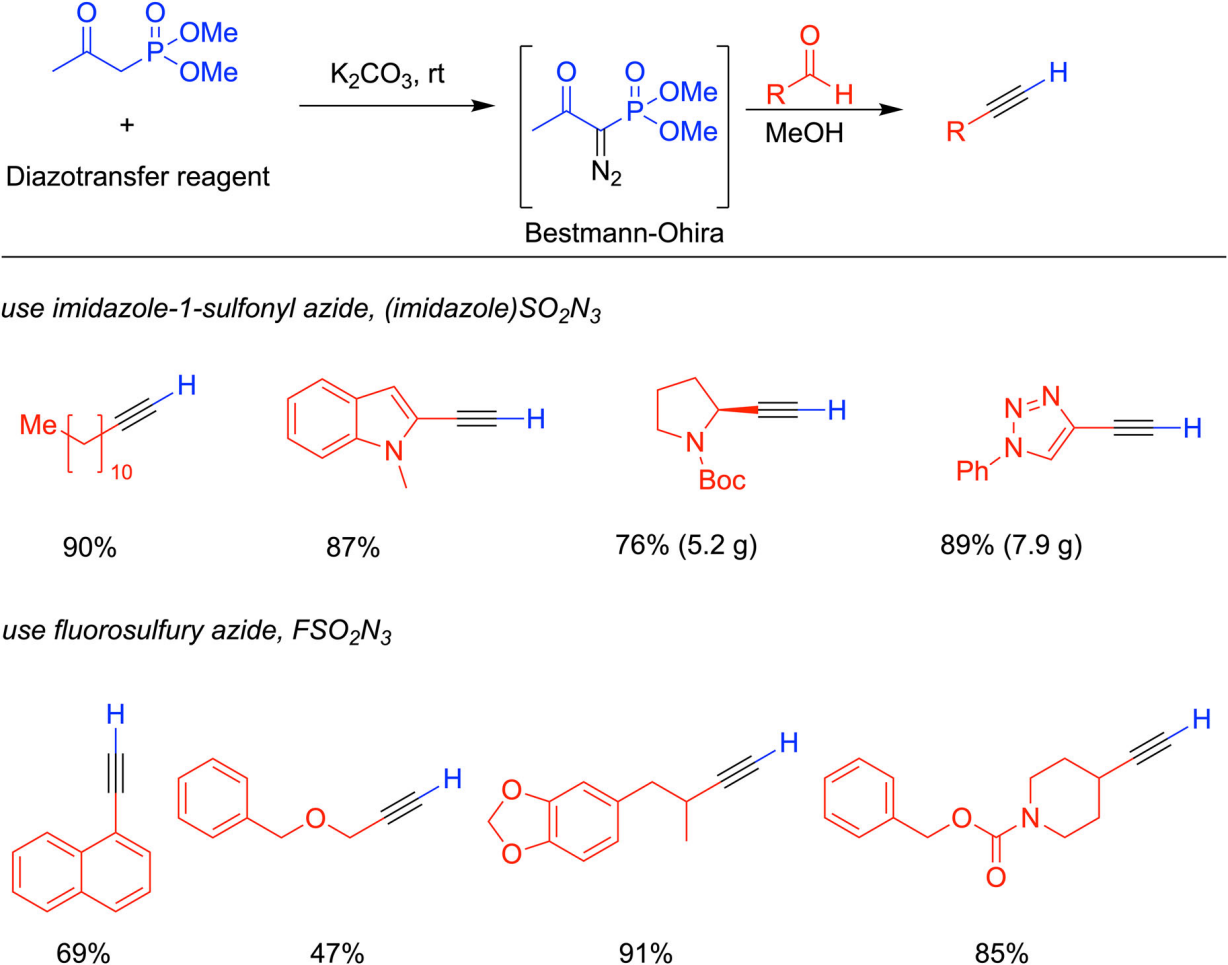
Selected examples of in situ generation of Bestmann‐Ohira reagent to make alkynes.

While the synthesis improves upon the Seyferth‐Gilbert reaction with its milder conditions, preparing the Bestmann‐Ohira reagent originally utilized tosyl azide, causing some practitioners to question the safety of scaling up the alkynylation reaction. This problem could be addressed by employing bench‐stable diazo transfer agents to form the Bestmann‐Ohira reagent in situ, which subsequently reacts with aldehydes to afford alkynes directly. Both imidazole‐1‐sulfonyl azide^[^ ^48^ ^]^ and fluorosulfury azide^[^ ^49^ ^]^ have been demonstrated to serve as effective and stable diazo‐transfer reagents, enabling the one‐pot conversion of aldehydes into terminal alkynes under mild conditions. The method utilizing imidazole‐1‐sulfonyl azide can be performed on a gram scale. Representative examples of these two strategies are summarized in Scheme 10.

It is also noteworthy that the Bestmann‐Ohira reaction can be conducted in tandem with other reactions in a one‐pot procedure. These include using esters and Weinreb amides initiated with reducing reagents like DIBAL‐H^[^ ^50^ ^]^ to generate the desired aldehyde starting material or starting from alcohols by first conducting an oxidation with MnO_2_ followed by in situ treatment under Bestmann‐Ohira conditions.^[^ ^51^ ^]^

### 3.4 More Recent Advances in Bestmann‐Ohira‐Type Reactions With Ph_3_PCN_2_

**Scheme 11:**
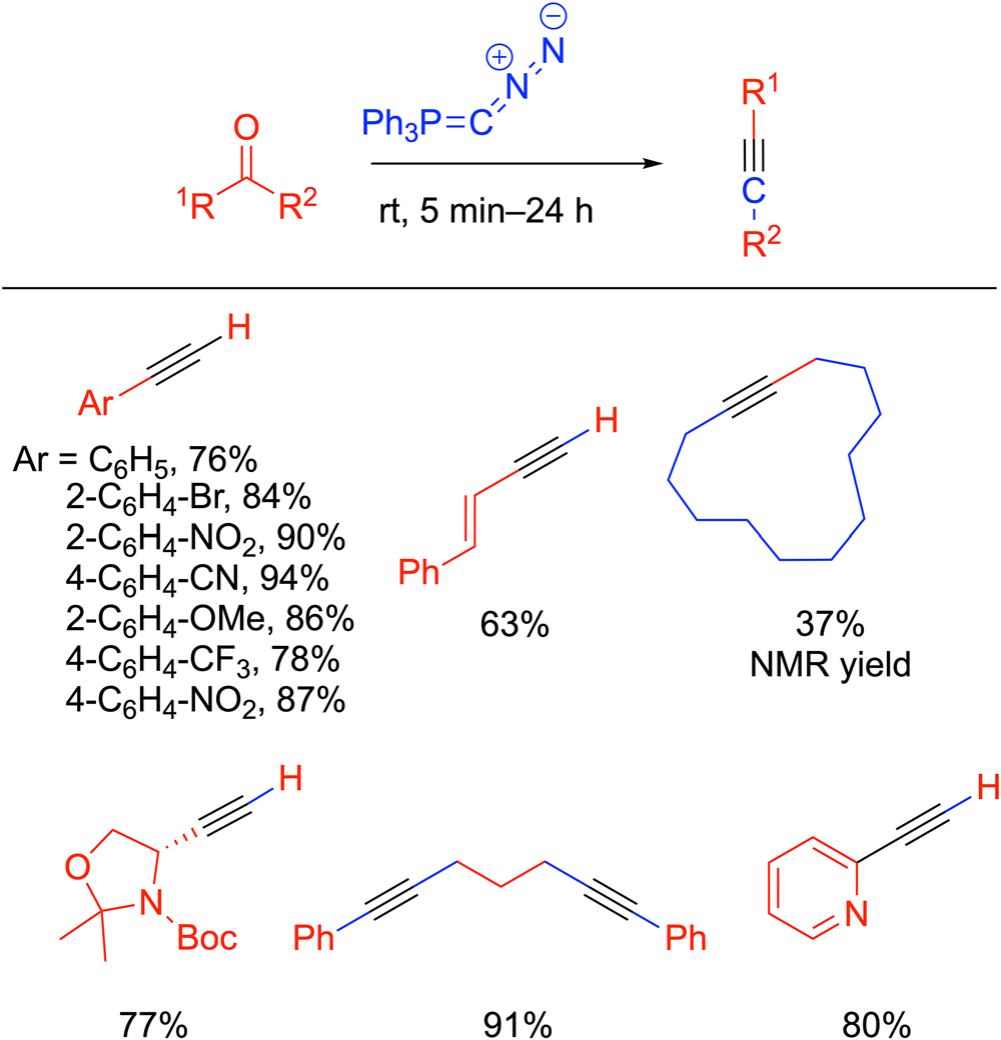
Alkyne synthesis with Ph_3_PCN_2_ and selected examples.

Hansmann and coworkers^[^ ^52^ ^]^ sought to find alternative C‐atom transfer reagents due to the limited range of those that were available (Scheme 11). Inspired by the Seyferth‐Gilbert and Bestmann‐Ohira reagents, which eliminate phosphate and dinitrogen in the conversion of aldehydes into alkynes, the group synthesized Ph_3_PCN_2_. The interesting feature of this diazophosphorous ylide is its formal charge of 0 on the carbon stabilized by triphenylphosphine and dinitrogen and its bent P─C─N bond angle [121.6°]. These workers envisioned that with both N_2_ and PPh_3_’s lability, the reagent could perform elimination similar to that of the Bestmann‐Ohira reagents. Interestingly, Ph_3_PCN_2_ shows remarkable thermal stability under an inert atmosphere, with decomposition at a temperature of roughly 150 °C. The reagent was also stable under prolonged heating in solution at 100° C. Unlike the Bestmann‐Ohira reagent, which requires the use of a protic solvent such as methanol, Ph_3_PCN_2_ can be used in benzene, toluene, and acetonitrile.

When Ph_3_PCN_2_ was reacted with aldehydes or ketones, the corresponding alkynes were formed. In the proposed mechanism, the first step is a Wittig‐type reaction to give a diazoalkane, R^1^R^2^C═C═N_2_, followed by loss of N_2_ and generation of the vinylidene (R^1^R^2^C═C:) and a 1,2‐shift, as previously outlined in Scheme 9. The reactions with aldehydes and ketones occurred at room temperature, and many substrates reacted in as little as 5 minutes. The reaction with Ph_3_PCN_2_ tolerated various functional groups. For example, aryl aldehydes with electron‐withdrawing and electron‐donating substituents and sterically bulky substrates all showed high yields. Ketone substrates reacted to give diaryl, dialkynes, and cyclic alkynes. Selected examples are shown in Scheme 11.

The advantage of this approach is that it avoids azide transfer reagents used in the synthesis of the Bestmann‐Ohira reagent. The reagent Ph_3_PCN_2_ is synthesized^[^ ^53^ ^]^ from Ph_3_P═C═PPh_3_ and N_2_O and was used as generated (without removal of the first equiv Ph_3_PO). Drawbacks to this method include the use of N_2_O gas, the generation of Ph_3_PO, and the 37% overall yield in generation of Ph_3_PCN_2_. Time will tell if this interesting synthesis of alkynes and enynes is adopted by the community and if a more user‐friendly synthesis of the reagent can be developed.

### 3.5 Colvin Synthesis of Alkynes

**Scheme 12:**
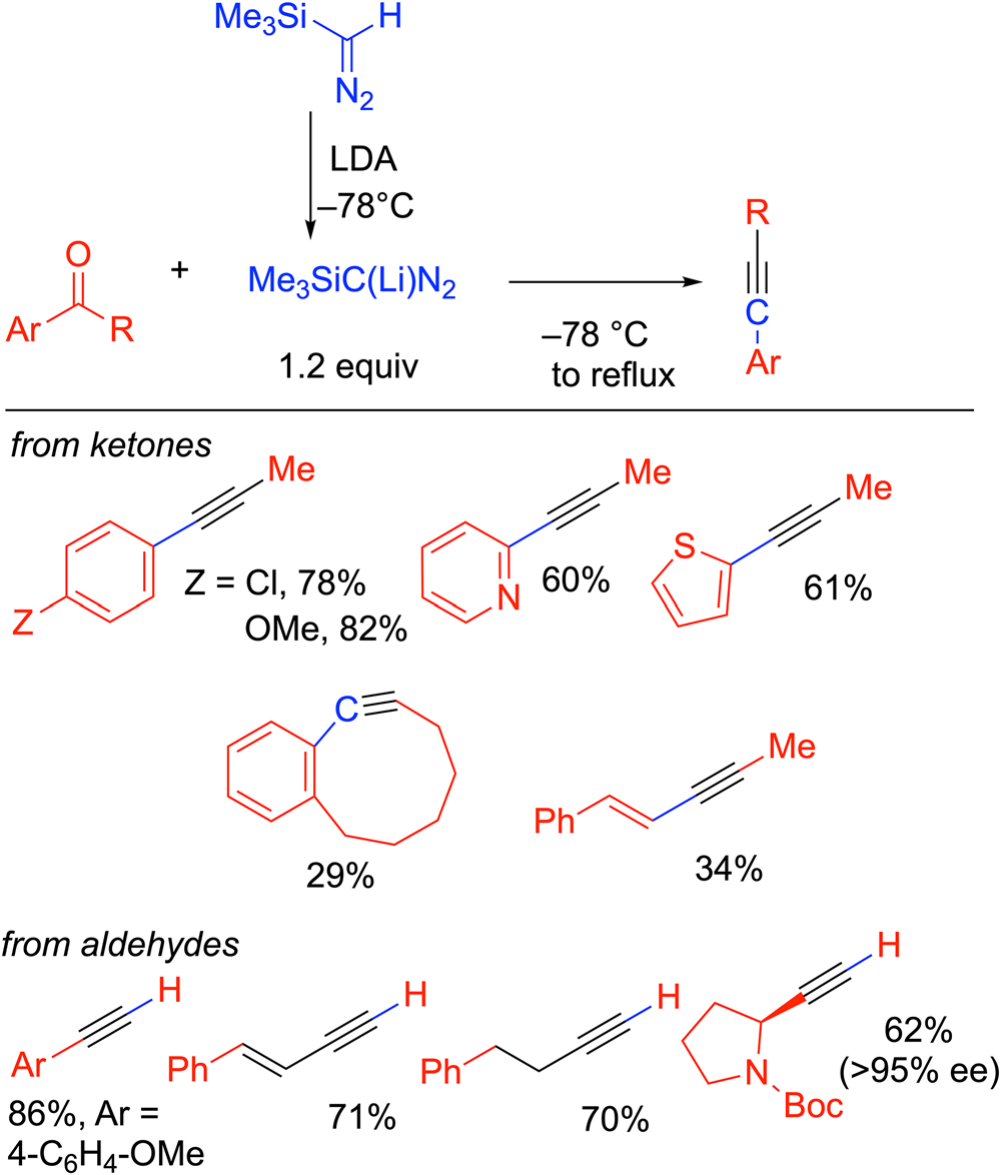
Colvin rearrangement with selected examples.

**Scheme 13:**
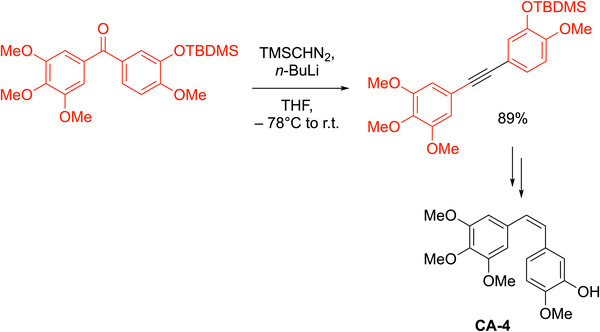
Application of the Colvin rearrangement to the synthesis of CA‐4.

Colvin^[^ ^34^ ^]^ and Hamill^[^ ^35^ ^]^ developed the reaction of lithium trimethylsilyldiazomethane, Li(Me_3_Si)C═N_2_ (Scheme 12), with ketones to form internal alkynes (3 examples). The scope of this process was dramatically improved by Aoyama, Shioiri, and coworkers.^[^ ^54^ ^]^ They demonstrated that treatment of TMSCHN_2_ with LDA at −78 °C in THF provided the lithiated intermediate, Li(Me_3_Si)C═N_2_ (Scheme 12). Slow addition of the carbonyl compound at −78 °C under argon gas with stirring for 1 hour was followed by heating under reflux for 3 hours and aqueous workup. The procedure was successful with aryl alkyl ketones (including those with heteroaryl groups) to form internal alkynes and even a benzocyclooctenone derivative for the preparation of a cyclic alkyne. Aldehydes formed terminal alkynes in moderate to high yields (50–80%). Substrates included benzaldehyde, cinnamyl aldehyde, dihydrocinnamaldehyde, and an enantioenriched proline derivative that underwent conversion to the alkyne without loss of enantiomeric excess. The Colvin reaction has been used in synthesis,^[^ ^55^ ^]^ notably in the preparation of combretastatin A‐4 (Scheme 13),^[^ ^56^ ^]^ but is not as widely used as the phosphorus‐based diazo methods. The Colvin rearrangement was also used to examine migratory aptitudes of the substituents on the carbonyl substrates, where it was confirmed that the relative rates of 1,2‐shift in alkylidene carbenes are H>>Ph>>alkyl. In the case of unsymmetrical benzophenone electrophiles, however, more electron‐rich aryl groups migrated faster, although the difference is nuanced.^[^ ^57^ ^]^

**Scheme 14:**
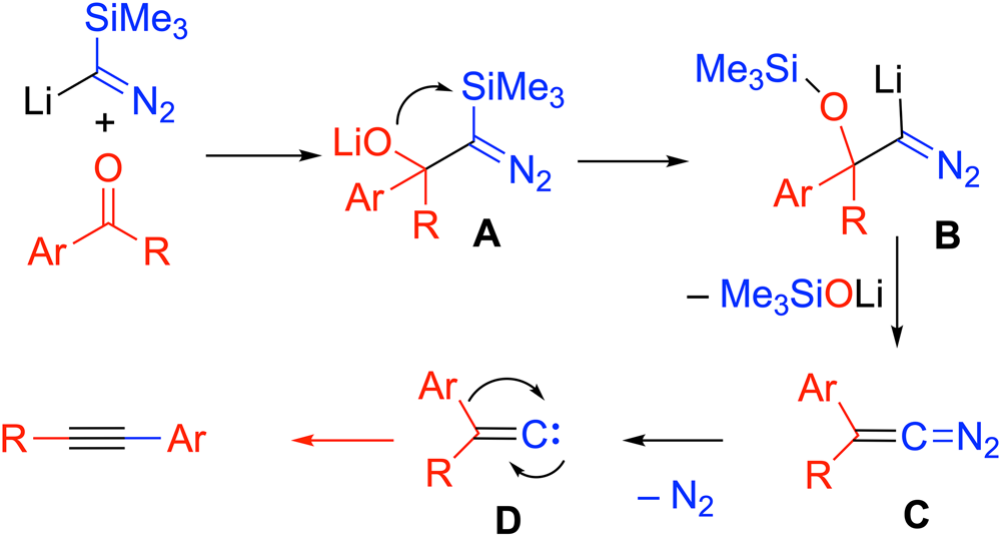
Mechanism of the Colvin alkynylation of aldehydes and ketones.

The mechanism of the Colvin alkyne synthesis involves deprotonation of the diazo derivative and addition to the carbonyl group to form a diazoalkoxide (**A**, Scheme 14). The alkoxy group then adds to the Si‐center in a [1,3]‐Brook rearrangement and undergoes elimination of Me_3_SiOLi. Loss of dinitrogen from **C** generates the vinylidene (**D**) that then triggers the rearrangement to afford the alkyne product.

## 4 Double‐Elimination Pathways

### 4.1 Double Elimination to Generate Diynes and Cyclooctenes

**Scheme 15:**
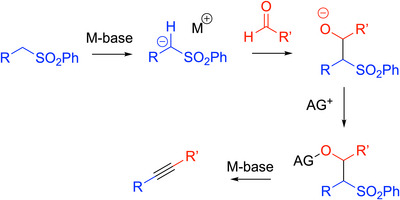
Otera's one‐pot strategy for the formation of triple bonds.

Otera and coworkers reported several versatile addition/double elimination strategies to generate alkynes using sulfone and aldehyde coupling partners (Scheme 15).^[^ ^58^, ^59^, ^60^, ^61^ ^]^ The plan was that deprotonation of the sulfone with base (*n‐*BuLi) at low temperature (−78 °C) and addition of the aldehyde would form the first C─C bond. The activating groups (AGs), often TMS‐Cl, Ac_2_O, and sometimes ClPO(OEt)_2_, are added at low temperature to the alkoxide intermediate. The double elimination is performed with bases, including KO^t^Bu, LDA, and LiN(SiMe_3_)_2_ (1.2 to 10 equiv), often at −78 °C.

**Scheme 16:**
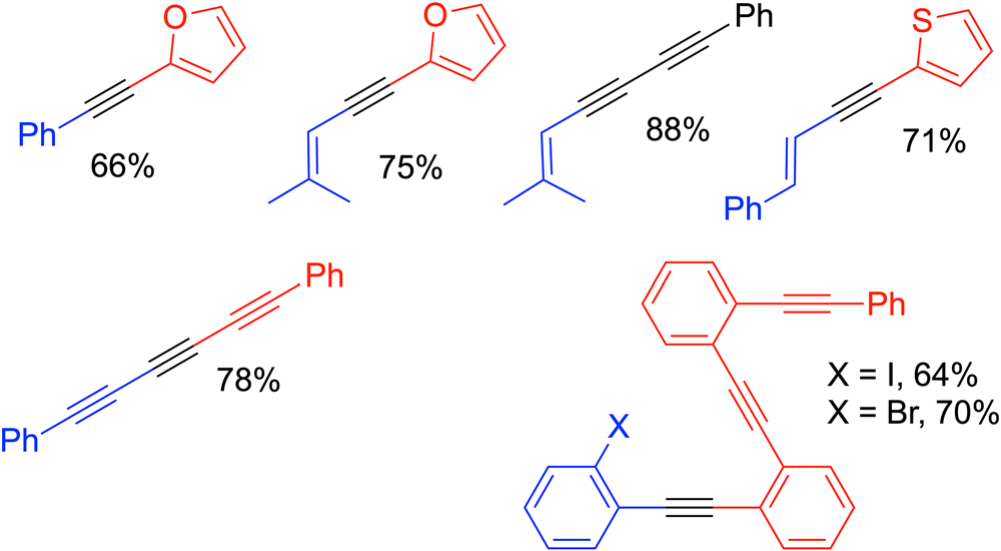
Selected examples from the Otera alkynylation of aldehydes.

The Otera reaction is fairly general, providing diaryl alkynes, conjugated enynes, and poly‐ynes in moderate to very good yields, as exemplified in Scheme 16. With the use of aliphatic aldehydes and sulfones, elimination can lead to mixtures of allenes and alkynes.

**Scheme 17:**
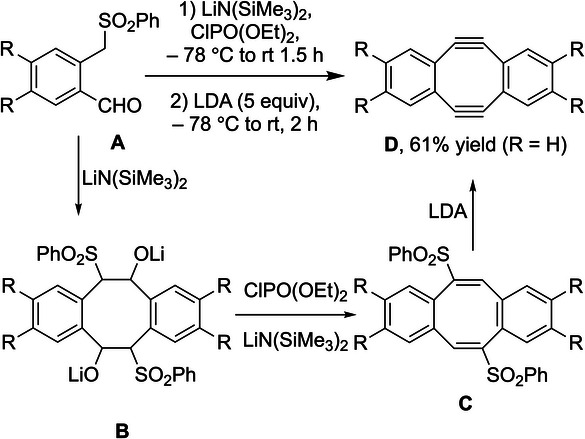
Synthesis of strained diynes from 2‐formyl benzylic sulfones.

Otera and coworkers applied their one‐pot alkyne synthesis to a beautiful preparation of 5,6,11,12‐tetradehydrodibenzo[*a*,*e*]cyclooctenes (**D**, Scheme 17),^[^ ^62^ ^]^ which are highly strained compounds that are often used for bioconjugation through click chemistry^[^ ^63^ ^]^ and construction of macromolecules through Diels‐Alder reactions.^[^ ^64^, ^65^ ^]^ In this work, the formyl sulfone was first deprotonated by LiN(SiMe_3_)_2_ (2 equiv based on **A**) at −78 °C and then underwent intermolecular condensation with the formyl group of another molecule of substrate to form a saturated eight‐membered ring (**B**, Scheme 17). Intermediate **B** was trapped in situ by the AG, diethylchlorophosphate in this case, and then eliminated by the excess LiN(SiMe_3_)_2_ to form the vinyl sulfone. Upon treatment with LDA (5 equiv), the vinyl sulfone underwent a second elimination to form the product. The reaction is impressive given the increase in strain going from the saturated eight‐membered ring **B** to **C** and then the product **D** (calculated to be 81.8 kcal/mol uphill, as reflected in the bent acetylenic bonds (155.7°). All steps need to be done under cryogenic temperatures. In a more recent work, Orita, Otera, and coworkers reported the synthesis of more substituted diynes using the same approach, although most were performed with isolation of the bis(sulfone) intermediate.^[^ ^66^ ^]^

The Otera synthesis is valuable due to its simplicity and high tolerance to different types of alkyne products, including diynes and cyclic alkynes, which are hard to access with other methods. However, the usage of strong bases, like LDA, and cryogenic temperatures limit its application to smaller scales.

### 4.2 Diethyl Phosphorochloridate

1‐Alkynylphosphonates are typically synthesized by reacting halogenated phosphates with deprotonated terminal alkynes.^[^ ^67^ ^]^ This approach is limited, however, by its narrow substrate scope and moderate yields. An improved one‐pot strategy for the synthesis of 1‐alkynylphosphonates employs diethyl trichloromethylphosphonate, a reagent that has proven its use in organic synthesis.^[^ ^68^, ^69^ ^]^ Based on their previous work with this reagent,^[^ ^70^ ^],[^ ^71^ ^]^ Dizière and Savignac sought to extend the utility of diethyl trichloromethylphosphonate to make 1‐alkynylphosphonates in a one‐pot procedure.^[^ ^72^ ^]^

**Scheme 18:**
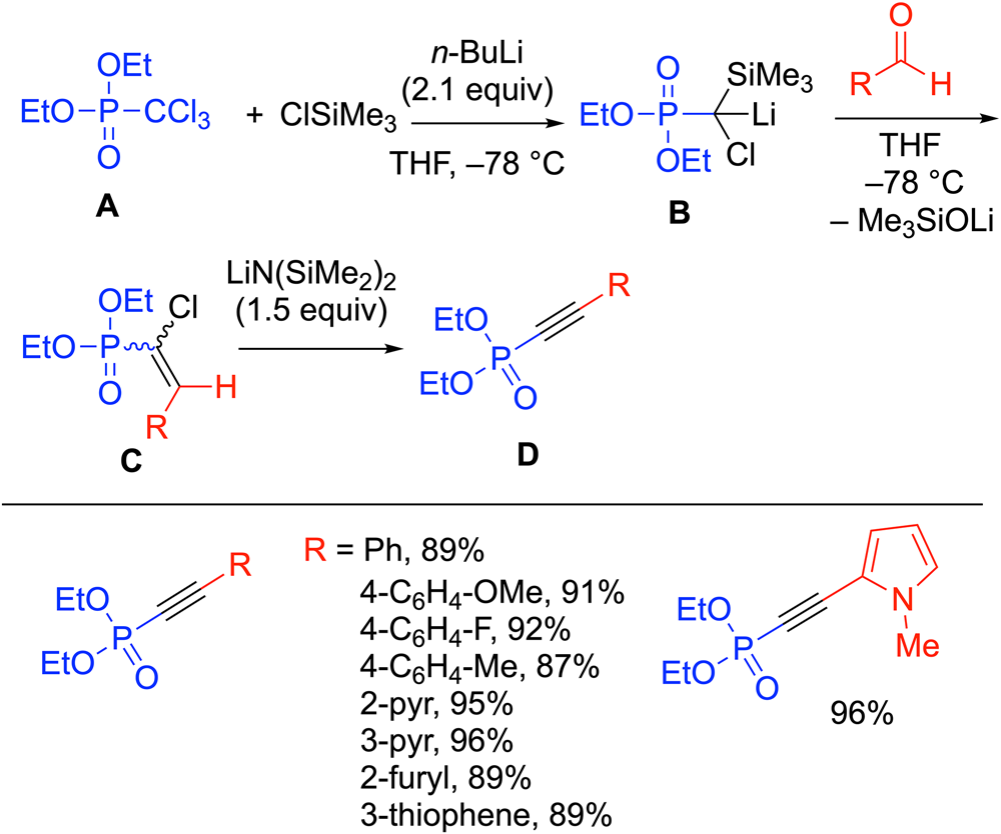
1‐Alkynylphosphonate synthesis from diethyl trichloromethylphosphonate.

First, diethyl trichloromethylphosphonate and chlorotrimethylsilane are mixed with butyllithium (2.1 equiv) in THF, resulting in a double chlorine‐lithium exchange (Scheme 18). The exchange forms a stabilized silylated carbanion **B**, which can be trapped by an aldehyde to form an alkoxide intermediate that decomposes via a Peterson elimination to form alkene **C**. LiN(SiMe_3_)_2_ was chosen as the base (1.5 equiv) for the elimination (**C→D**) due to its steric hindrance and reduced nucleophilicity, which minimizes side products derived from addition to the electrophilic triple bond. HN(SiMe_3_)_2_ is also easily removed upon acidic workup. The reaction was found to have high yields (87–96%) with substituted benzaldehydes and heteroaryl aldehyde derivatives (Scheme 18).

The procedure to synthesize 1‐alknylphosphonates utilizing diethyl trichloromethylphosphonate provides a high‐yielding and facile method of producing alkynes without a transition‐metal catalyst. Overall, the procedure highlights the power of applying phosphonates as a reagent to participate in one‐pot, metal‐free alkyne syntheses.

### 4.3 Two‐Carbon Homologation for the Synthesis of Difluoromethyl Alkynes

**Scheme 19:**
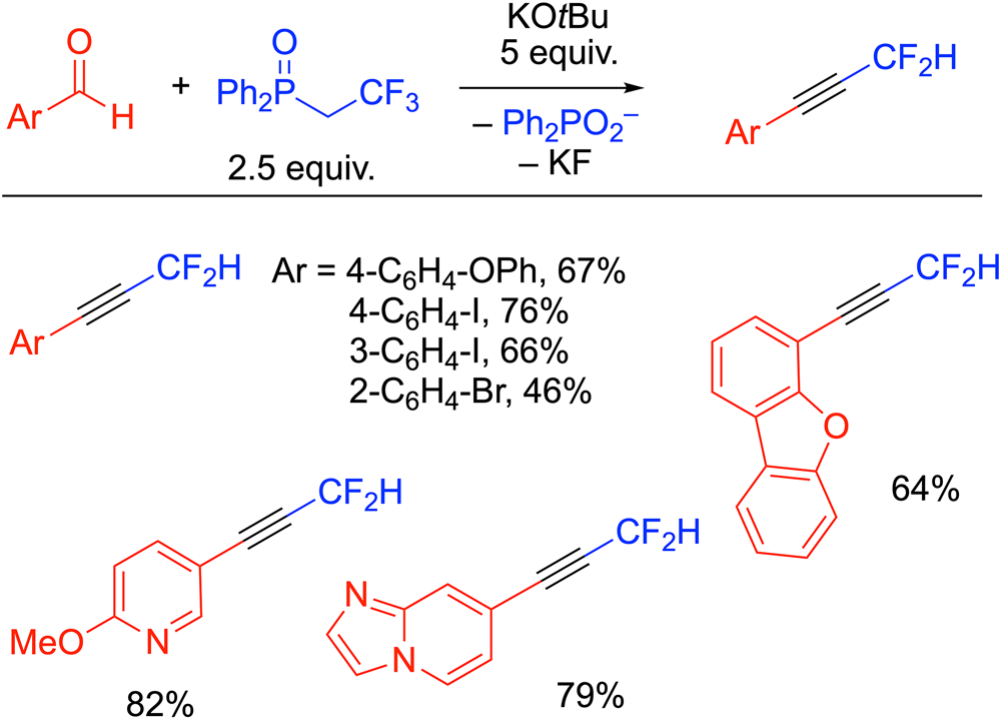
Two‐carbon homologation for the synthesis of difluoromethyl alkynes.

The difluoromethyl group is finding increased utility in medicinal chemistry, and, as discussed earlier, alkynes are commonly used precursors for the preparation of heterocycles. Zhu, Chu, and coworkers^[^ ^73^ ^]^ developed a one‐pot two‐carbon homologation reaction for the transformation of benzaldehyde derivatives into aryl difluoromethyl alkynes under relatively mild conditions (Scheme 19). Beginning with the known diphenyl(2,2,2‐trifluoroethyl)phosphine oxide, Ph_2_P(═O)CH_2_CF_3_ (2.5 equiv), which is readily prepared on a gram scale,^[^ ^74^ ^]^ treatment with KO^t^Bu (5 equiv.) and aryl aldehydes for 10 minutes at rt generated the difluoromethyl alkynes. The scope of the reaction was very good, working with electron‐rich and electron‐poor aldehydes as well as those bearing heterocyclic groups.

**Scheme 20:**
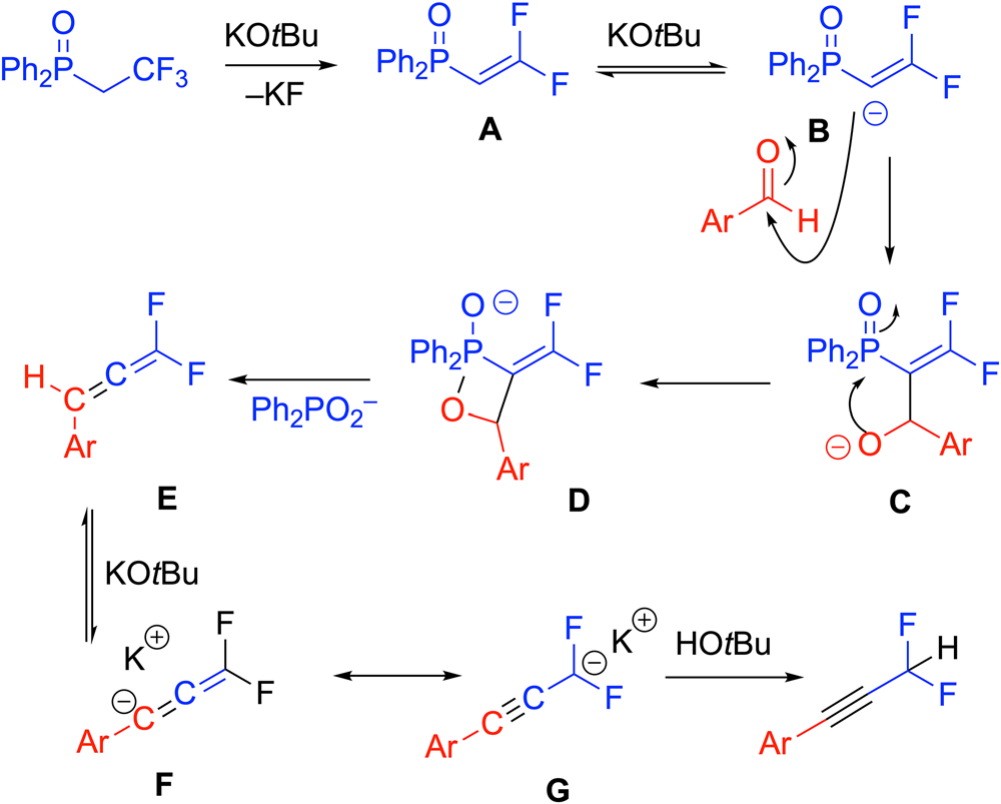
Proposed mechanism for the two‐carbon homologation.

Based on literature precedence and deuterium labeling studies, the authors proposed that the initial steps involve deprotonation of the Ph_2_P(═O)CH_2_CF_3_ by the KO^t^Bu, and elimination of KF to generate the unsaturated intermediate vinyl phosphine oxide **A** (Scheme 20). Intermediate **A** is deprotonated by the KO^t^Bu and the resulting anion **B** reacts with the aldehyde substrate to generate addition product **C**. **C** forms the oxaphosphetane intermediate **D** and then undergoes a Horner–Wadsworth–Emmons‐type elimination to generate the gem‐difluoroallene, **E**. The allene was not reported to be observed but appears to undergo base‐promoted isomerization to the observed alkyne.

Drawbacks to this procedure include the basic conditions that do not permit the use of aliphatic aldehydes and no known scaleup examples. On the whole, however, the procedure is mild and efficient, providing difluoromethyl alkyne building blocks in an expedient fashion.

### 4.4 Benzotriazoles

**Scheme 21:**
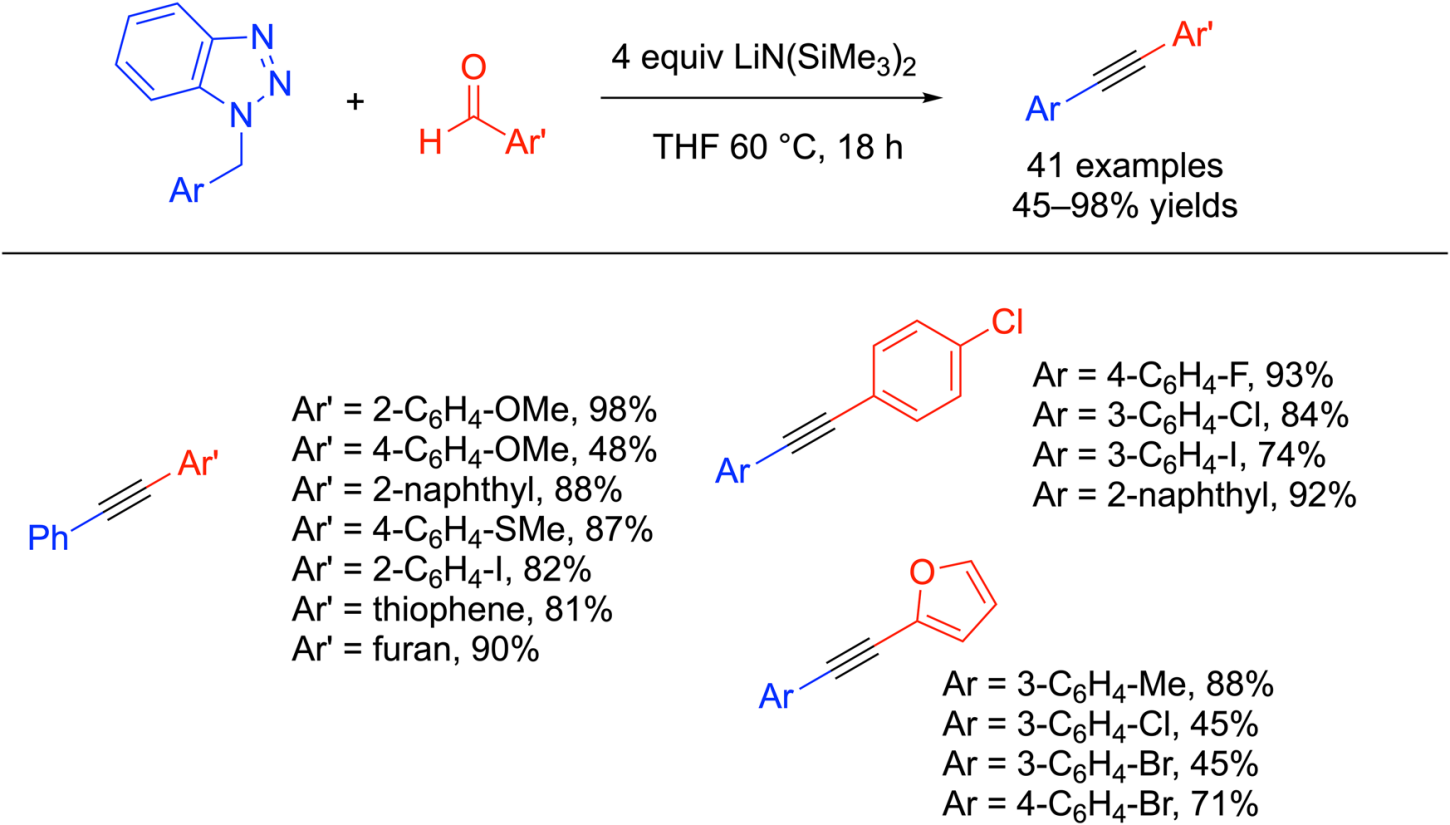
Synthesis and scope of 1,2‐diaryl alkynes from benzotriazoles and aldehydes.

**Scheme 22:**
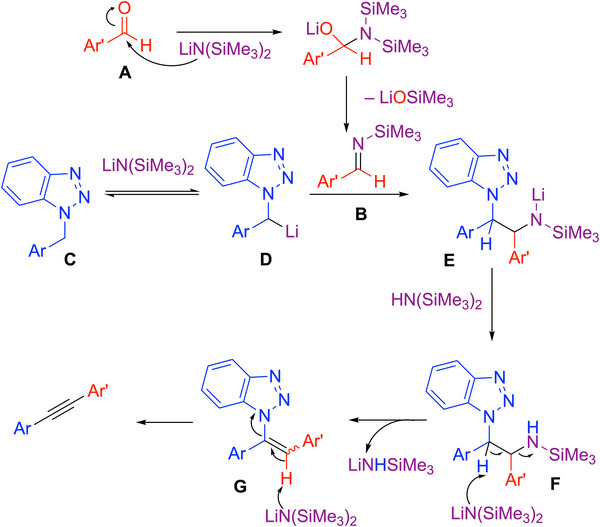
The Proposed mechanism for the generation 1,2‐diaryl alkynes from benzotriazoles and aldehydes.

Katritzky^[^ ^75^, ^76^, ^77^ ^]^ and Hay^[^ ^78^ ^]^ and their coworkers demonstrated that *N*‐benzyl benzotriazoles could react with imines through Mannich‐type reactions to make alkynes over multiple steps. Based on this observation, Chen and coworkers developed a one‐pot double elimination process using benzyl benzotriazoles and aldehydes (Scheme 21).^[^ ^79^ ^]^ In this work, the *N*‐(trimethylsilyl)imines were generated in situ from aldehydes upon treatment of the benzaldehyde derivative with LiN(SiMe_3_)_2_ (as outlined below, Scheme 22). The imine then reacted with the *N*‐benzyl benzotriazole through a double elimination to generate the alkyne product. Overall, 41 examples of 1,2‐diaryl alkynes were reported in 45–98% yields. Selected examples are shown in Scheme 21.

The mechanism of the reaction in Scheme 21 is outlined in Scheme 22. The aldehyde (**A**) underwent addition with LiN(SiMe_3_)_2_ to generate an *N*‐TMS imine (**B**) in situ through an aza‐Peterson reaction.^[^ ^80^, ^81^ ^]^ The benzyl benzotriazole (**C**) was deprotonated by LiN(SiMe_3_)_2_ to give organolithium (**D**) that reacted with the imine (**B**) to generate the amide intermediate (**E**) through a Mannich‐type addition. Under the basic conditions, the proton transferred to the amide **E** sets up the first elimination via **F**. The excess LiN(SiMe_3_)_2_ promotes the second elimination from **G** to expel the benzotriazinide and generate the alkyne product.

This is an effective method to make 1,2‐diaryl alkynes, avoiding the use of harsh conditions and additives. The broad substrate scope of the aldehyde exemplifies the reaction's high tolerance. However, the need for benzyl benzotriazoles to be prepared separately is a drawback. One cannot help but wonder if the reaction could be made catalytic in the presence of benzyl halide to regenerate the benzyl benzotriazole.

### 4.5 Organocatalytic Synthesis of Alkynes

**Scheme 23:**
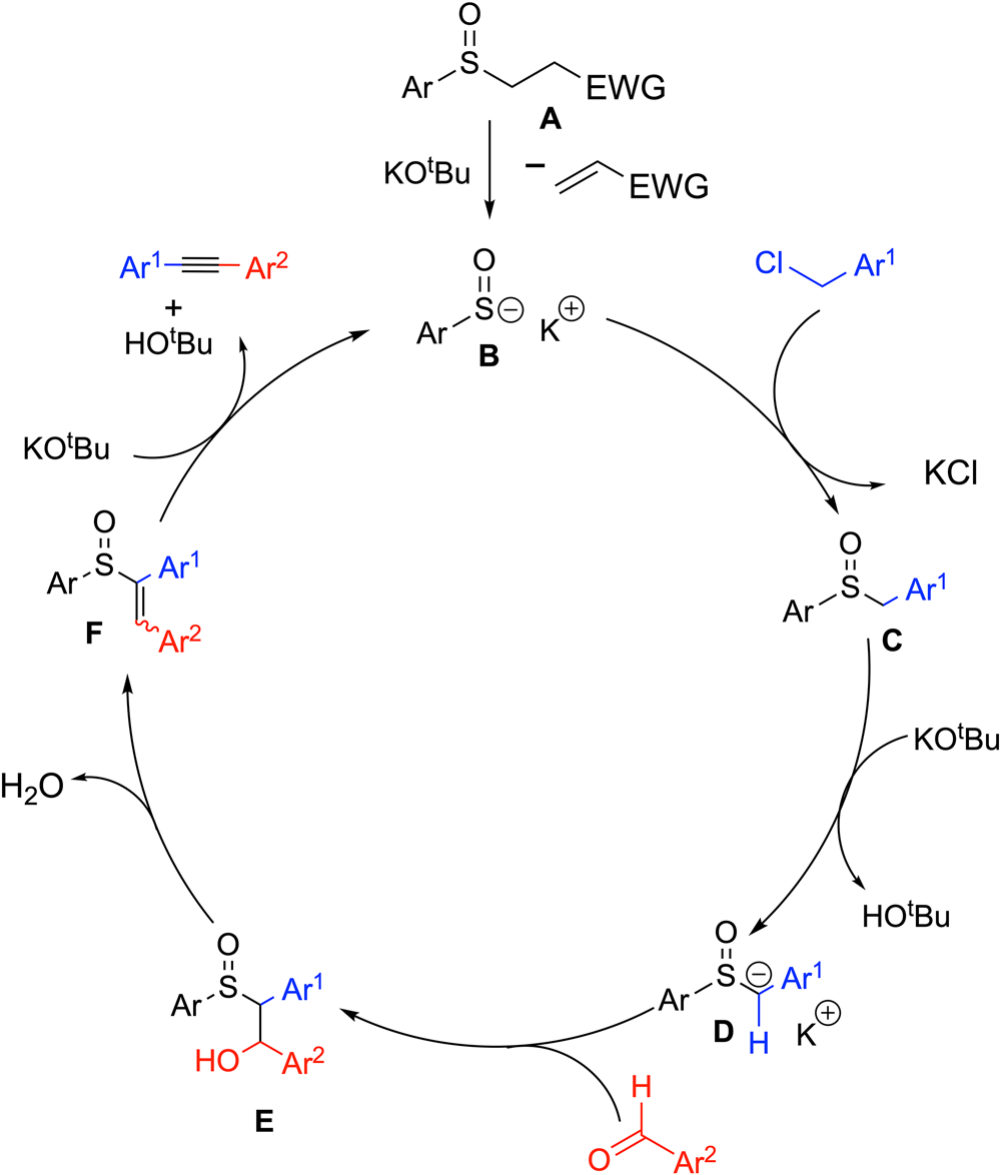
Catalytic cycle for the sulfenate anion‐catalyzed generation of 1,2‐diaryl alkynes.

Walsh and coworkers developed the first organocatalytic method to prepare 1,2‐diaryl alkynes from aryl aldehydes and benzylic halides.^[^ ^82^ ^]^ The key species in this reaction is the sulfenate anion (ArSO^−^) which is both a good nucleophile and a good leaving group and has been applied in the catalytic syntheses of *trans*‐stilbenes^[^ ^83^ ^]^ and aziridines.^[^ ^84^, ^85^ ^]^ As shown in Scheme 23, beginning from sulfoxide **A** (EWG═Ph), a base‐promoted elimination generates the sulfenate anion (ArSO^–^, **B**). The sulfenate anion first reacts with the benzyl chloride via an S_N_2 to generate a benzylic sulfoxide, PhSOCH_2_Ph (**C**). Under basic conditions with KO^t^Bu, the benzylic sulfoxide (p*K_a_* = 27 in DMSO^[^ ^86^ ^]^) undergoes reversible deprotonation to give an intermediate that resembles an enolate (**D**) and reacts similarly. The deprotonated sulfoxide then undergoes an aldol‐like reaction with the aryl aldehyde to give an aldol‐type product (**E**). Base‐mediated elimination of water generates the vinyl sulfoxide (**F**), which undergoes a second E2 elimination, this time of the sulfenate anion catalyst, and forms the third bond of the alkyne.

**Scheme 24:**
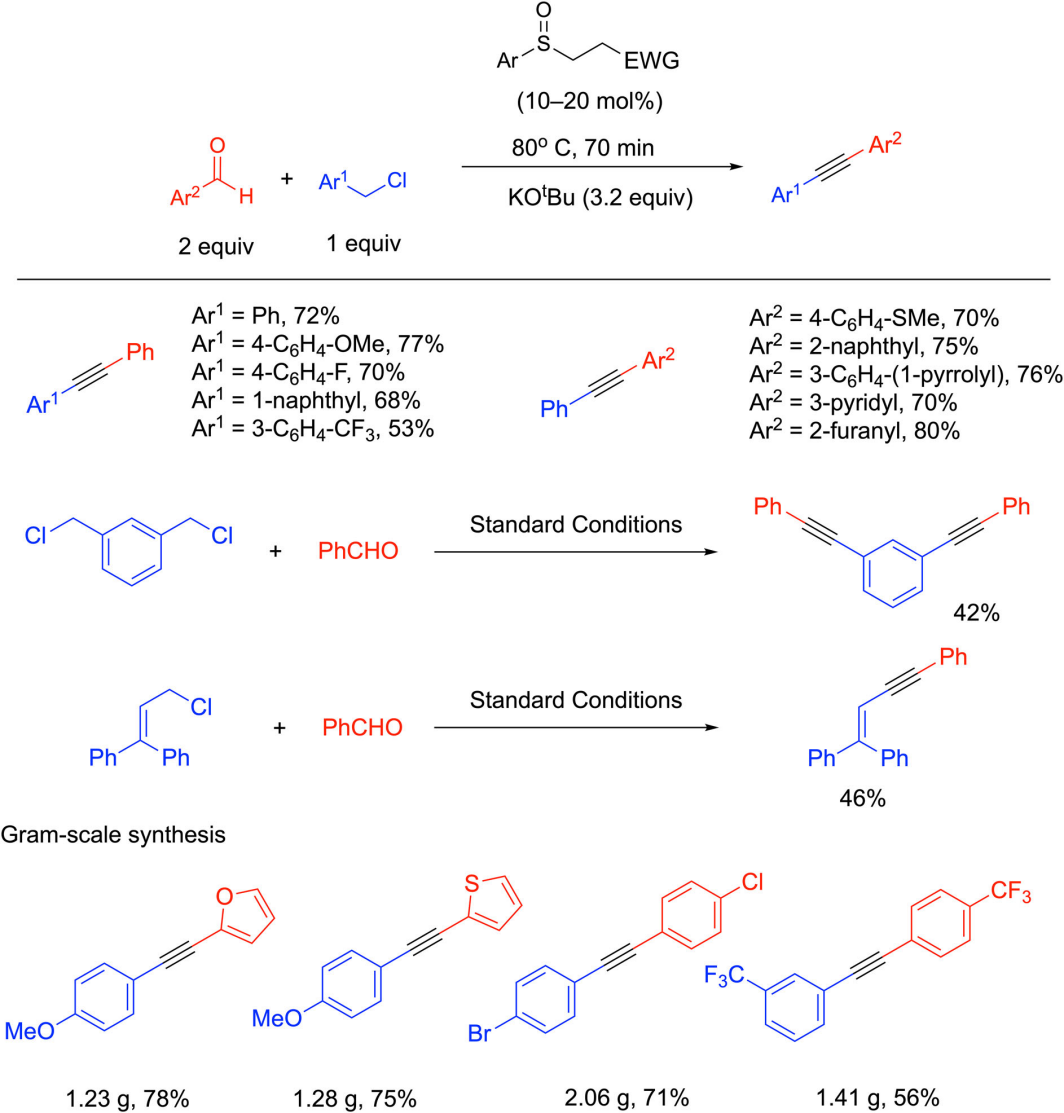
Scope of the sulfenate anion catalyzed synthesis of alkynes.

Different groups on both aryl aldehydes and benzylic halides were employed, and the method showed good tolerance of functional groups. In total, 31 examples were reported in 42–80% yields, including diyne and enyne products. Selected examples are shown in Scheme 24. Reactions performed on a gram scale were done with 10 mol% precatalyst.

This synthesis is attractive for several reasons. First, this method is the only example that generates all three bonds of an alkyne in one vessel under organocatalytic conditions. Instead of transition‐metal catalysts (like in the Sonogashira coupling), an organocatalyst, sulfenate anion, was used, which circumvents potential issues from metal residues. Meanwhile, this method avoids harsh reagents and utilizes readily available chemicals. The authors developed a broad substrate scope and applied this method to gram‐scale syntheses (Scheme 24, with 10 mol% catalyst loading). The drawbacks are the precatalyst (sulfoxide) takes several steps to prepare, and the reaction requires slow addition of the benzyl chloride to limit the direct benzylation of the KO^t^Bu. The main byproduct is derived from the Cannizzaro reaction, which is why 2 equiv aldehyde was used. Presumably other organocatalysts that operate through similar mechanisms can be introduced.

### 4.6 1,2‐Diaryl Alkyne Synthesis

1,2‐Diaryl acetylenes are a class of alkynes that have demonstrated bioactivities in various pharmacological applications, including anti‐inflammatory^[^ ^87^ ^]^ and antiproliferative agents.^[^ ^88^ ^]^ Several FDA‐approved medications feature 1,2‐diaryl alkyne backbones, such as trazarotene,^[^ ^89^ ^]^ olverembatinib,^[^ ^90^ ^]^ and futibatinib^[^ ^91^ ^]^ (Figure 1). The most common way to prepare 1,2‐diaryl alkynes is to functionalize terminal alkynes through Sonogashira cross‐coupling.^[^ ^92^ ^]^ However, as discussed previously, the Sonogashira procedure has its drawbacks, including the high cost of transition metal catalysts and phosphine ligands, the toxicity of copper salts, and the potentially difficult purification of the diaryl alkyne from the side products.^[^ ^93^, ^94^, ^95^ ^]^

**Scheme 25:**
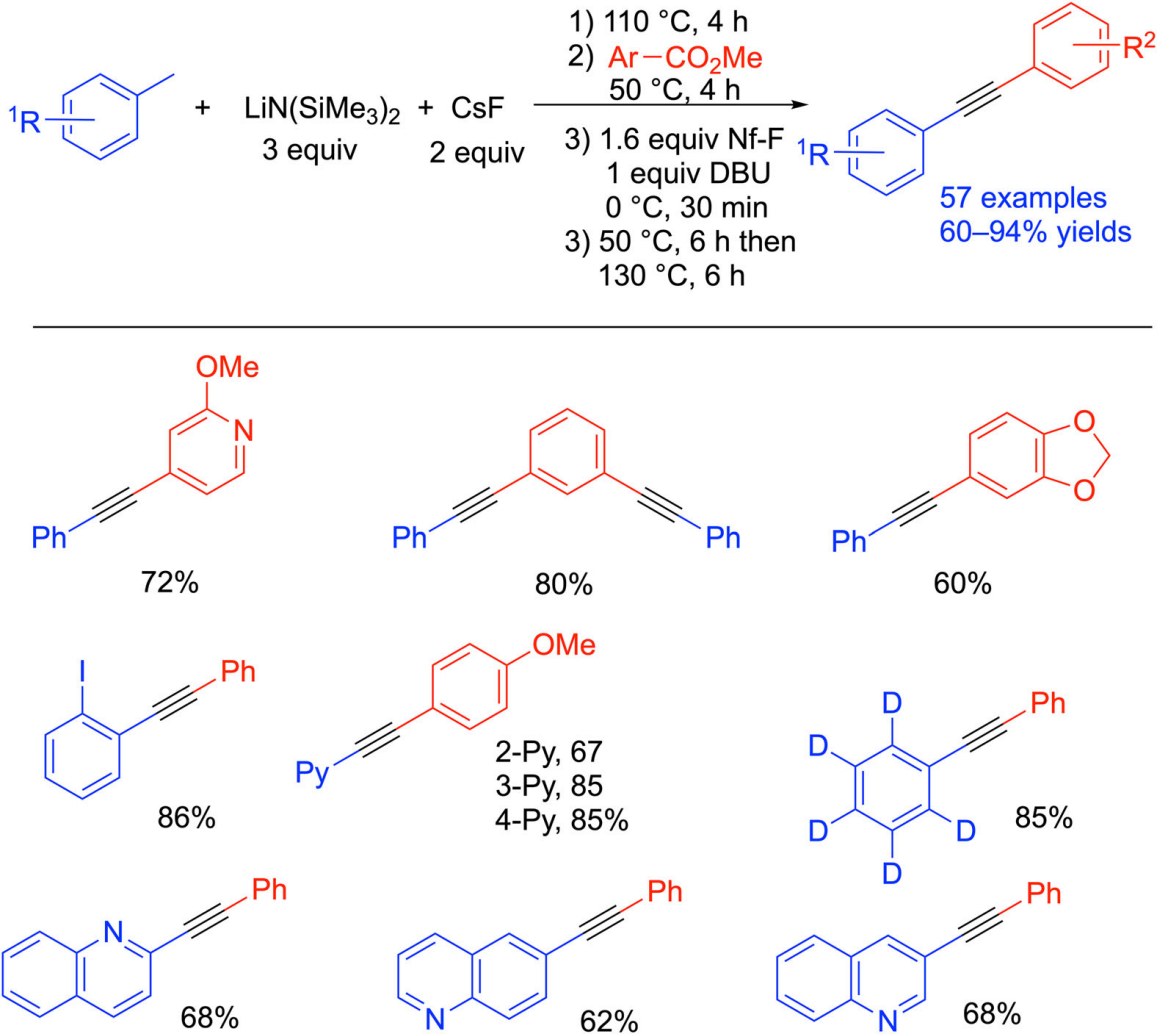
Synthesis and scope of 1,2‐diaryl alkynes from toluene derivatives and methyl benzoates.

Mao, Walsh, and coworkers developed a one‐pot synthesis of diaryl alkynes that utilizes readily available chemical feedstocks, like toluenes and methyl benzoates, and circumvents the use of transition‐metal catalysts (Scheme 25).^[^ ^96^ ^]^ The synthesis started with the preheating of LiN(SiMe_3_)_2_ and CsF with toluene derivatives at 110 °C, and then the addition of methyl benzoate and an activating group, Nf─F (Nf─F = F─SO_2_CF_2_CF_2_CF_2_CF_3_). TBS─Cl and PhMe_2_SiCl were also used but had slightly lower yields. The activated enolate was eliminated by DBU to form the alkyne product. This method shows high tolerance of different functional groups on toluenes and methyl benzoates. Overall, 57 examples of 1,2‐diaryl alkynes were reported in 60–94% yields.

**Scheme 26:**
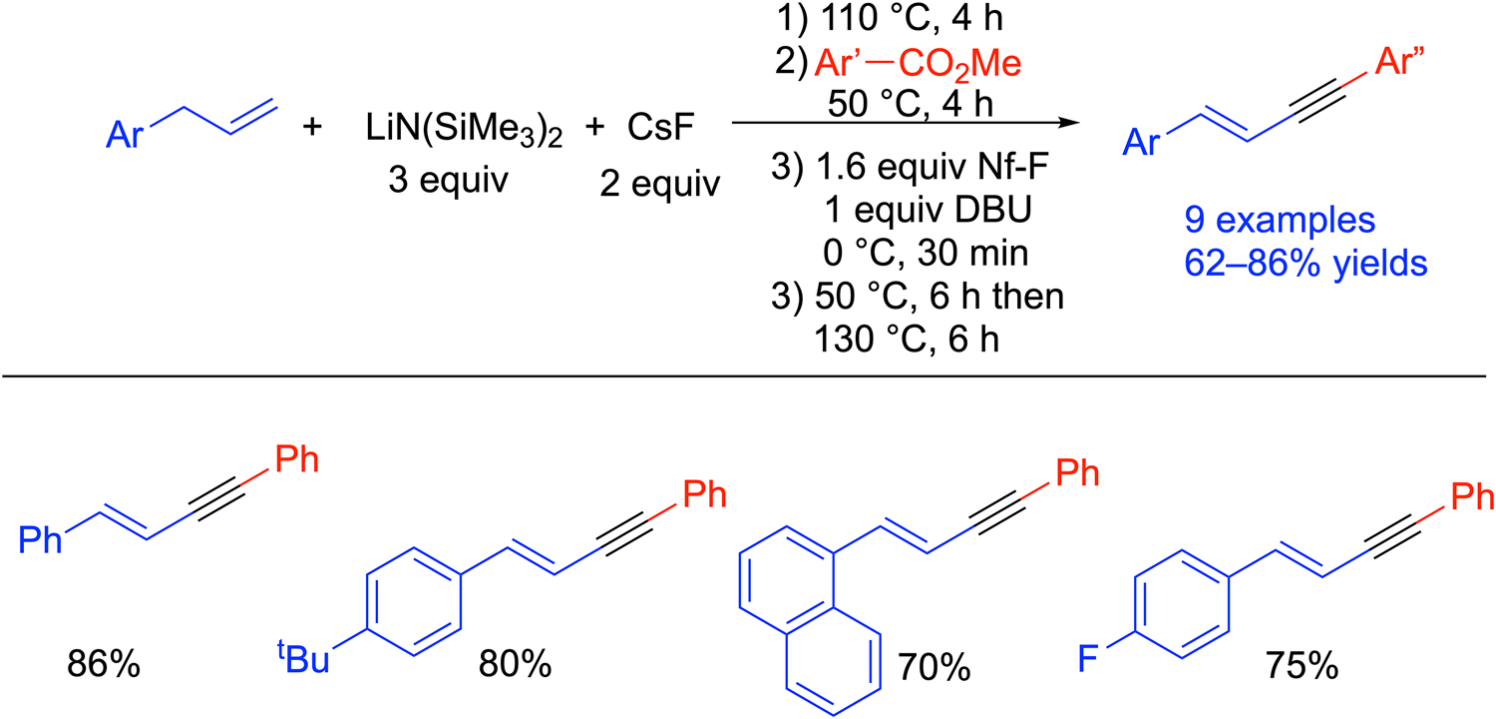
Synthesis and scope of enynes from allyl benzene and methyl benzoates.

In addition to toluenes, allyl benzenes were also used as pro‐nucleophiles in this method, and 9 examples of enynes were reported in 62–86% yields in this one‐pot procedure. Selected examples are shown in Scheme 26.^[^ ^96^ ^]^

**Scheme 27:**
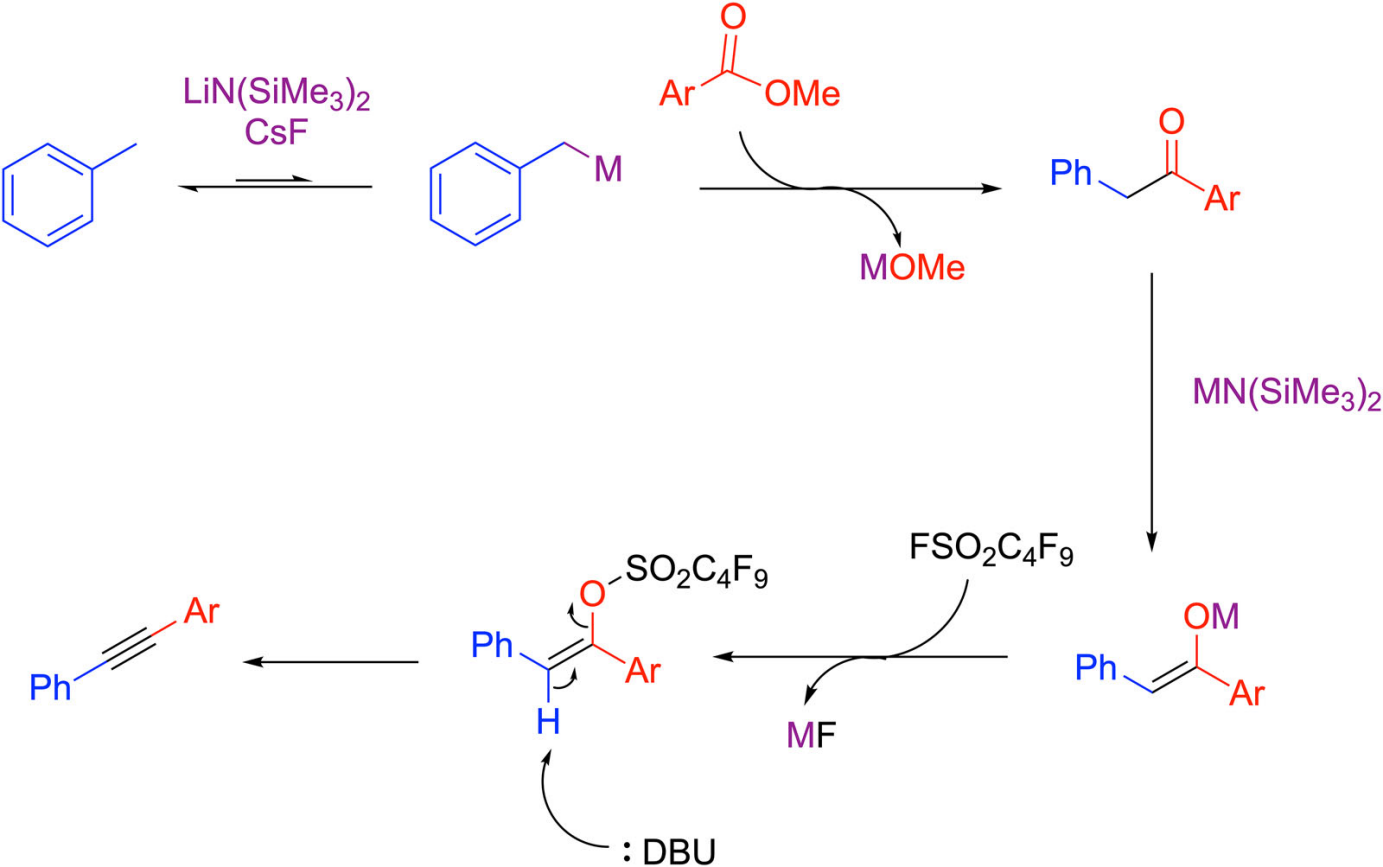
Proposed mechanism of reaction of toluene and methyl benzoate to form 1,2‐diaryl alkynes.

A proposed mechanism is outlined in Scheme 27. For coupling partners like weakly acidic toluenes, the active base was generated by preheating with LiN(SiMe_3_)_2_ and CsF at 110 °C in the pro‐nucleophile solvent. The authors postulated that a bimetallic silyl amide base system formed in the preheating step, which most likely generates either CsN(SiMe_3_)_2_ or mixed metal amides such as CsLi[N(SiMe_3_)_2_]_2_. It was envisioned that these bases would be capable of reversibly deprotonating the weakly acidic pro‐nucleophiles, including toluene (p*K_a_* = 43 in DMSO^[^ ^86^ ^]^). This deprotonation was facilitated by cation‐π interactions between the toluene derivative and the main group metal cation.^[^ ^97^, ^98^, ^99^ ^]^ The in situ formed benzylic organometallic then reacted with the methyl benzoate to form a ketone, which was rapidly deprotonated by excess MN(SiMe_3_)_2_ to generate an enolate. The enolate was transformed into an activated enolate by addition of Nf─F (Nf = SO_2_CF_2_CF_2_CF_2_CF_3_). The activated enolate was then eliminated by DBU and formed the alkyne product.

This synthesis is preferable due to its inexpensive materials (toluenes and methyl benzoates). By avoiding transition‐metal catalysts, stronger bases, and cryogenic temperatures, the procedure is much more attractive for scaling up. Unlike most of the procedures that have been developed starting from aldehydes, which are sensitive to oxidation and must be purified, this work utilizes more stable esters. A downside to the reaction is the activation step to form the active base, which involves heating of reagents in solvent at 110 °C with the pro‐nucleophile as solvent. With more acidic pronucleophiles, like methylated azaarenes, 3 equiv of the pronucleophile was used in 2‐methyl THF. The elimination also requires successive heating at different temperatures, which complicates the procedures.

### 4.7 Smiles‐Rearrangement‐Based One‐Pot Synthesis of Diarylacetylenes

**Scheme 28:**
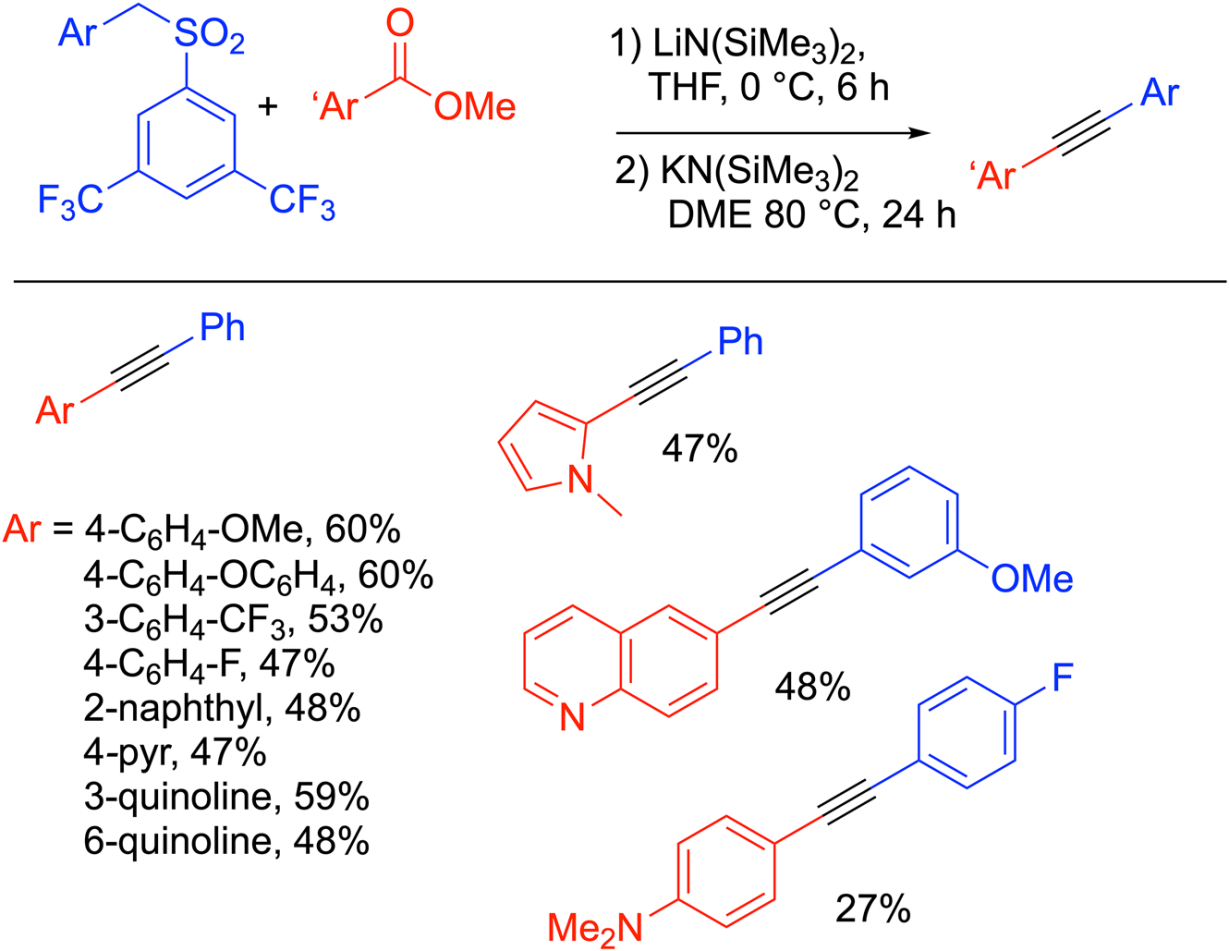
Smiles rearrangement route to alkynes.

One of the drawbacks of synthesizing diarylalkynes from toluenes and methyl benzoates, as shown in Scheme 25, is the activation of the enolate by conversion of the oxygen into a leaving group by addition of Nf─F or PhMe_2_SiCl. A streamlined version was envisioned by Walsh, Mao, and coworkers utilizing the Smiles rearrangement to generate a leaving group in situ. The idea used inexpensive methyl benzoate electrophiles and 1‐(benzylsulfonyl)‐3,5‐di(trifluoromethyl)benzene [ArCH_2_SO_2_(3,5‐C_6_H_3_─(CF_3_)_2_] pro‐nucleophiles under basic conditions to generate α‐keto‐sulfonate intermediates (Scheme 28).^[^ ^100^ ^]^ LiN(SiMe_3_)_2_ was first added to a mixture of methyl benzoate and the benzylic sulfone in THF under nitrogen at 0 °C, resulting in C─C bond formation and deprotonation. Afterwards, KN(SiMe_3_)_2_ and DME were added to the reaction mixture and stirred for 24 hours at 80 °C.

Under these optimized conditions, the team achieved diarylacetylenes in up to 69% yields. The reaction tolerated electron‐donating and electron‐withdrawing groups on the methyl benzoate, and heterocyclic diaryl alkynes were achievable with 27–60% yields using pyridine, quinoline, and *N*‐methylpyrrole as reactants. Likewise, the reaction tolerated a broad scope of substitutions on the 1‐(benzylsulfonyl)‐3,5‐di(trifluoromethyl)benzene in yields from 18% to 70%. Examples of product alkynes are shown in Scheme 28.

**Scheme 29:**
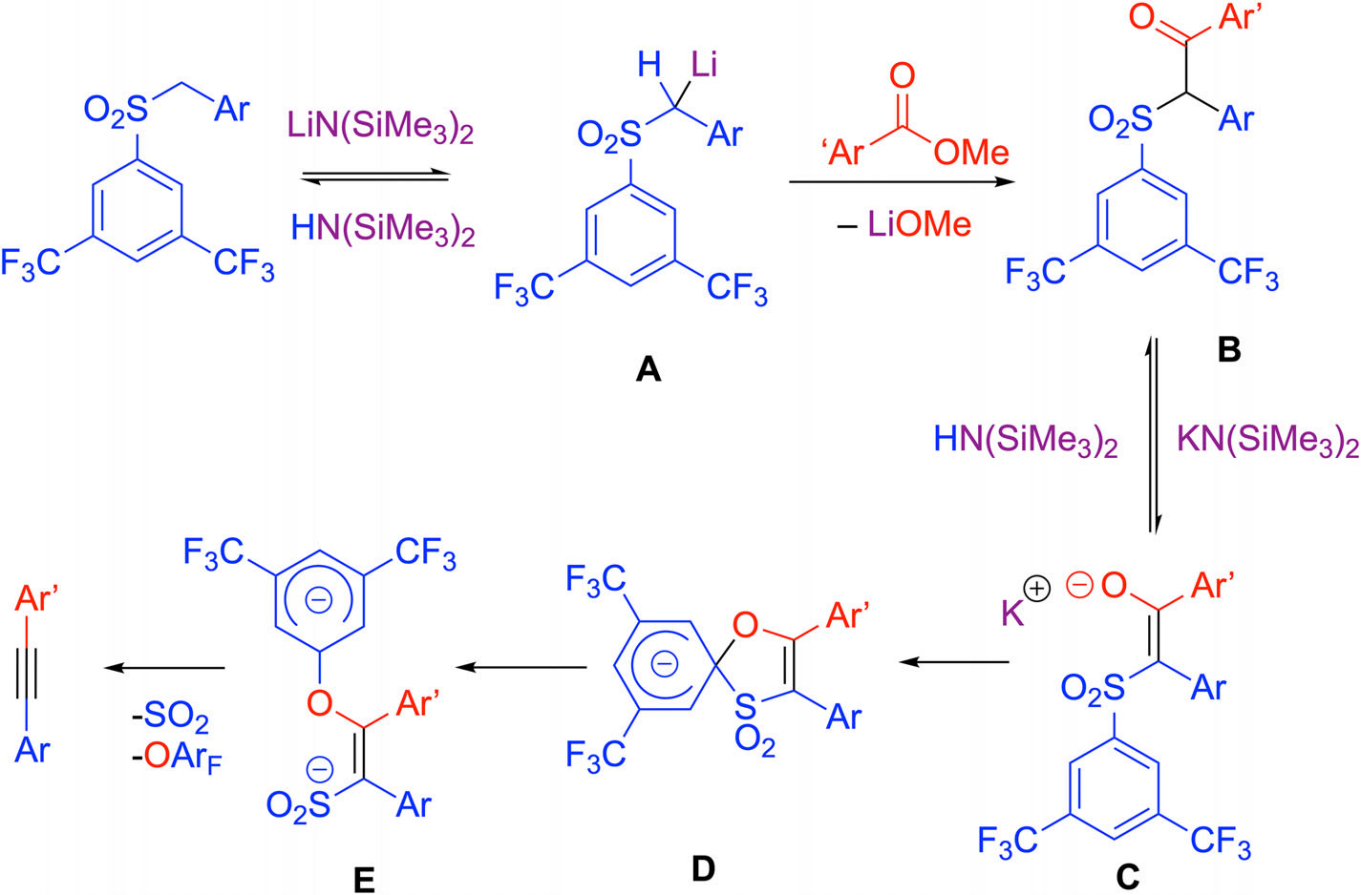
Mechanism to form alkynes through the Smiles rearrangement.^[^ ^100^ ^]^

The group proposed a mechanism showcased in Scheme 29. Reversible deprotonation of the benzylic sulfone by LiN(SiMe_3_)_2_ generates metallated **A**, which reacts with methyl benzoate to form the intermediate keto sulfone **B**. Intermediate **B** is then deprotonated by KN(SiMe_3_)_2_ to give the enolate **C**. With the K^+^ cation, the Smiles rearrangement can occur, driven by the loss of SO_2_ and aryloxide. The Smiles rearrangement was sluggish with the lithium base alone, which was proposed to interact more strongly with the enolate **C** and prevent the formation of **D**.

The procedure features a streamlined transition‐metal‐free route to diaryl alkynes using KN(SiMe_3_)_2_, methyl benzoates, and LiN(SiMe_3_)_2._ It is scalable, with gram‐scale syntheses that achieved 43–48% yields. A clear drawback of the synthesis is the low conversions. Overall, the paper highlights the utility of the Smiles rearrangement for the synthesis of alkynes in a one‐pot manner and opens the door for continued investigations to develop higher‐yielding processes.

### 4.8 Alkynyl Amide Synthesis

Among the family of alkynes, alkynyl amides are of special interest due to their presence in many organic molecules that show bioactivity.^[^ ^101^, ^102^, ^103^ ^]^ They also show high pharmaceutical potential, serving as a precursor for a quinoline‐based inhibitor.^[^ ^104^ ^]^ The prior syntheses of alkynyl amides generally elaborate existing alkynes and employ transition‐metal catalysts.^[^ ^105^, ^106^ ^]^ Qin and coworkers devised a transition‐metal‐free method using SO_2_F_2_ gas with secondary alcohols.^[^ ^107^ ^]^ However, this method is limited in scope, involving a toxic gas, and does not form all three bonds in one pot.

**Scheme 30:**
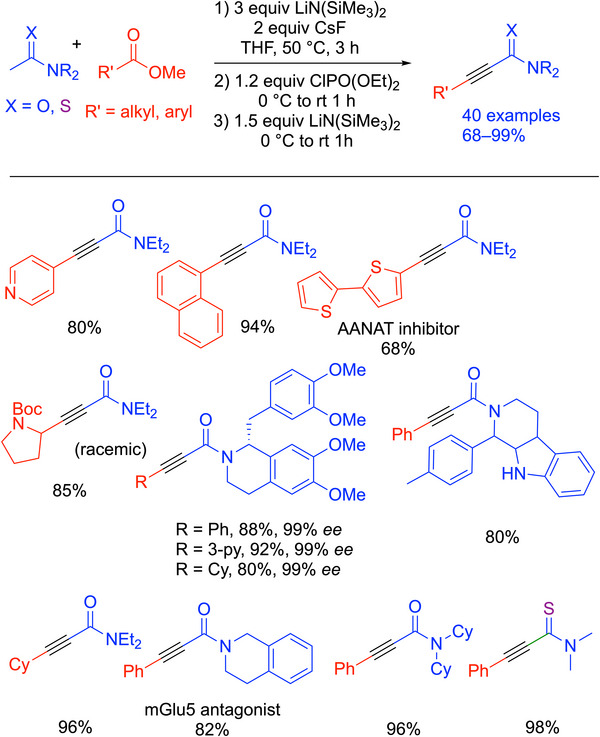
Synthesis and scope of alkynyl amides (Cy = cyclohexyl).

In recent work, Mao, Walsh, and coworkers developed a one‐pot synthesis of alkynyl amides that avoids these drawbacks (Scheme 30).^[^ ^108^ ^]^ Based on their one‐pot synthesis of 1,2‐diaryl alkynes (Scheme 25), a similar procedure to form alkynyl amides in a one‐pot, transition‐metal‐free fashion was introduced. Acetamides and methyl benzoates were first heated with LiN(SiMe_3_)_2_ and CsF at 50 °C. This resulted in an enolate that was then trapped by an activating group, AG, in this case diethyl chlorophosphate, to form an activated enolate. Elimination was performed with LiN(SiMe_3_)_2_ to generate alkynyl amides. This work demonstrated high tolerance on functional groups on the acetamides. This method could also be applied to both methyl benzoates and aliphatic esters to generate alkynyl amides with alkyl and aryl groups. A thioamide substrate was successfully converted to the thioamide‐based alkyne. Overall, 40 examples were reported in 68–99% yields. Selected examples are shown in Scheme 30. This method could be used to prepare enantioenriched alkynyl amides using enantioenriched amide precursors.

**Scheme 31:**
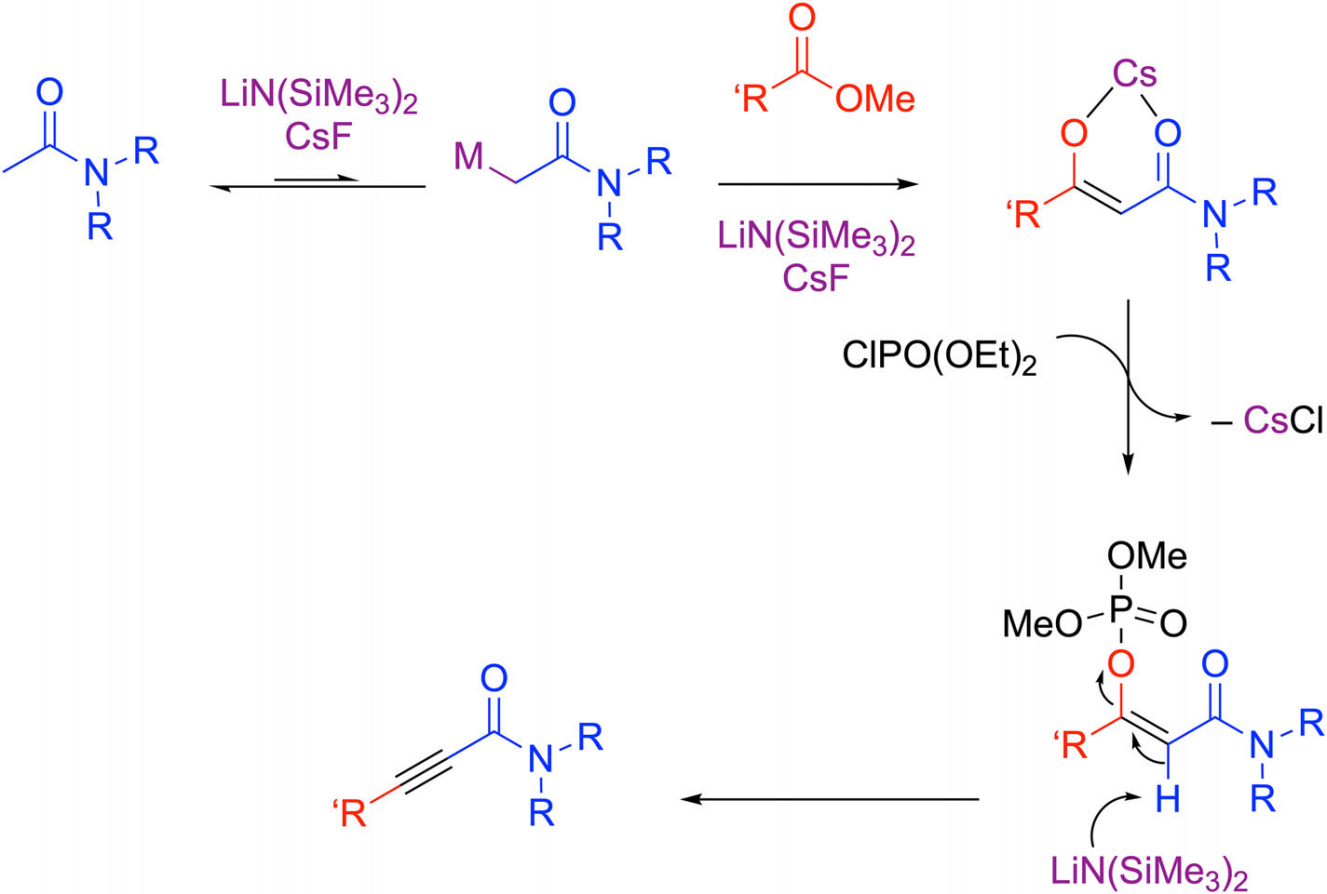
Proposed mechanism for the generation of alkynyl amides from acetamides and methyl benzoates.

The authors proposed a similar mechanism to their previous work (Scheme 31). The deprotonated acetamide and methyl benzoate underwent a Claisen condensation to generate the β‐dicarbonyl intermediates, which were rapidly deprotonated by the base. The intermediate was trapped by diethyl chlorophosphate to form an activated enolate. The authors found that the activation proceeded more effectively with the CsF additive. The activated intermediate was isolated and confirmed as the (Z)‐diastereomer and was eliminated by LiN(SiMe_3_)_2_ at 0 °C. It is interesting to note that when aliphatic esters were used, the deprotonation of the ester takes place due to its greater acidity than the amide coupling partner. However, the ester deprotonation is reversible and does not ultimately interfere with the productive amide deprotonation that leads to the Claisen condensation intermediate.

The synthesis is attractive due to its simple procedures and starting materials that are easy to access. No preheating was necessary due to the more acidic pronucleophiles (acetamides, p*K_a_* ∼35 in DMSO^[^ ^109^ ^]^) and the streamlined elimination with a phosphorus‐based AG. The mild conditions of this method enable the efficient and sustainable synthesis of a diverse range of alkynyl amides in a cost‐effective manner.

### 4.9 1‐Haloalkynes

#### 4.9.1 1‐Iodo Alkynes

**Scheme 32:**
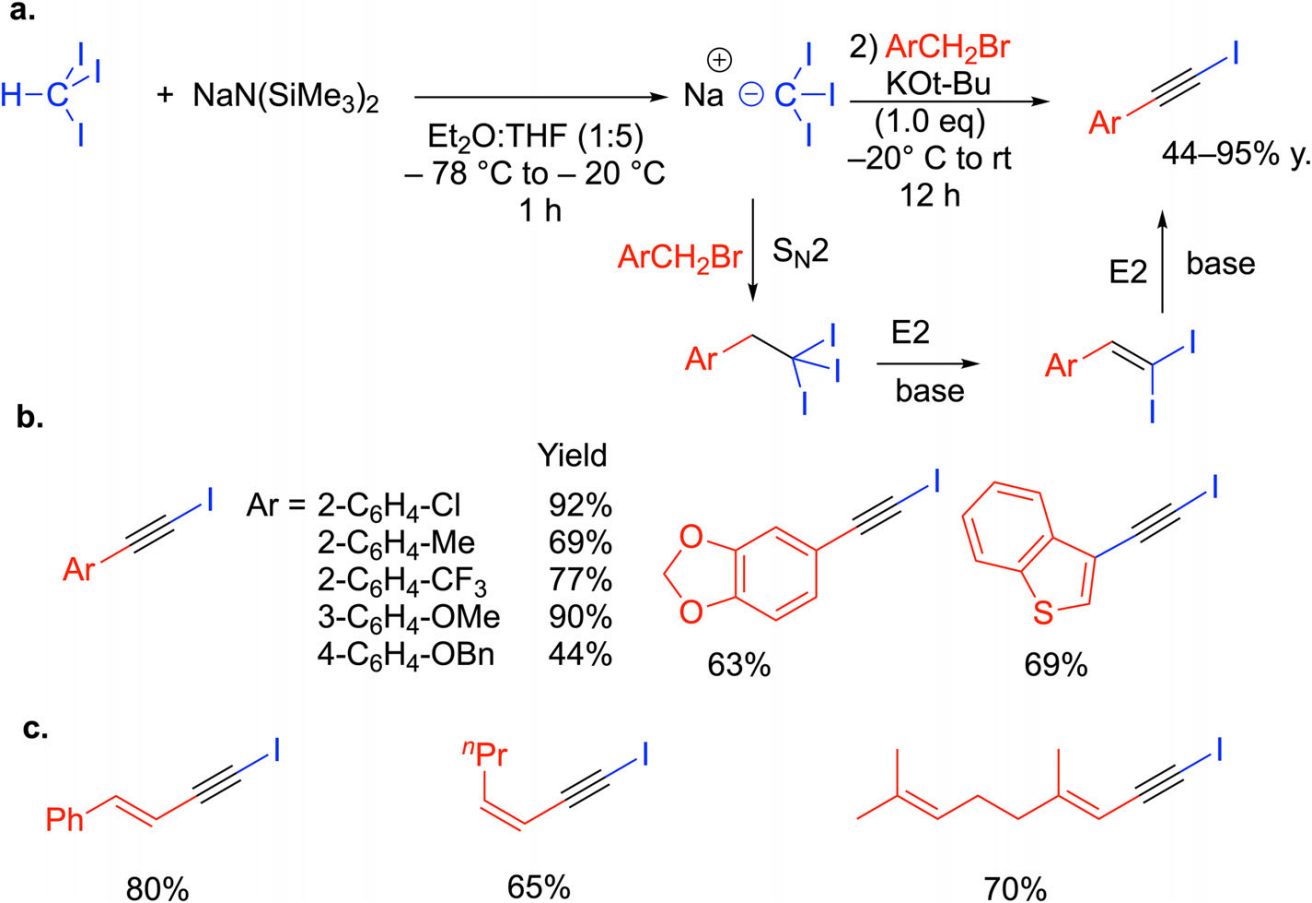
1‐Iodoalkynes from benzylic and allylic bromides via I_3_CNa.

Among alkynes, 1‐haloalkynes have proven to be versatile precursors for a variety of applications. 1‐Iodoalkynes are especially useful and have found applications in materials science, polymer chemistry, and organic synthesis. 1‐Iodoalkynes can be prepared by iodination of terminal alkynes in the presence of iodinating reagents and silver catalysts.^[^ ^110^, ^111^, ^112^ ^]^ A more efficient and economical one‐pot procedure for the synthesis of 1‐iodo alkynes was developed by Charette^[^ ^113^ ^]^ and coworkers that employs 1.5 equiv of CHI_3_ and 3 equiv of NaN(SiMe_3_)_2_. Combination of these reagents in the dark at −78 °C to −20 °C for 1 hour resulted in deprotonation of the iodoform to furnish the nucleophile, I_3_CNa. Addition of benzyl bromides resulted in S_N_2 to generate the triiodo ethyl benzene. Subsequent addition of 1 equiv of KO^t^Bu initiated a double elimination reaction to furnish 1‐iodoalkynes in 44–95% yields (Scheme 32a). Some examples are shown in Scheme 32b. Interestingly, the reaction could be extended to allylic bromides, which gave 1‐iodo enynes in 65–80% yields (Scheme 32c).

The strength of this work is its rapid preparation of functionalized 1‐iodoalkynes. The drawbacks are the need for cryogenic temperatures and the instability of the deprotonated iodoform, which is proposed to decompose by generating carbenes or carbenoids.

#### 4.9.2 1‐Bromo Alkynes

**Scheme 33:**
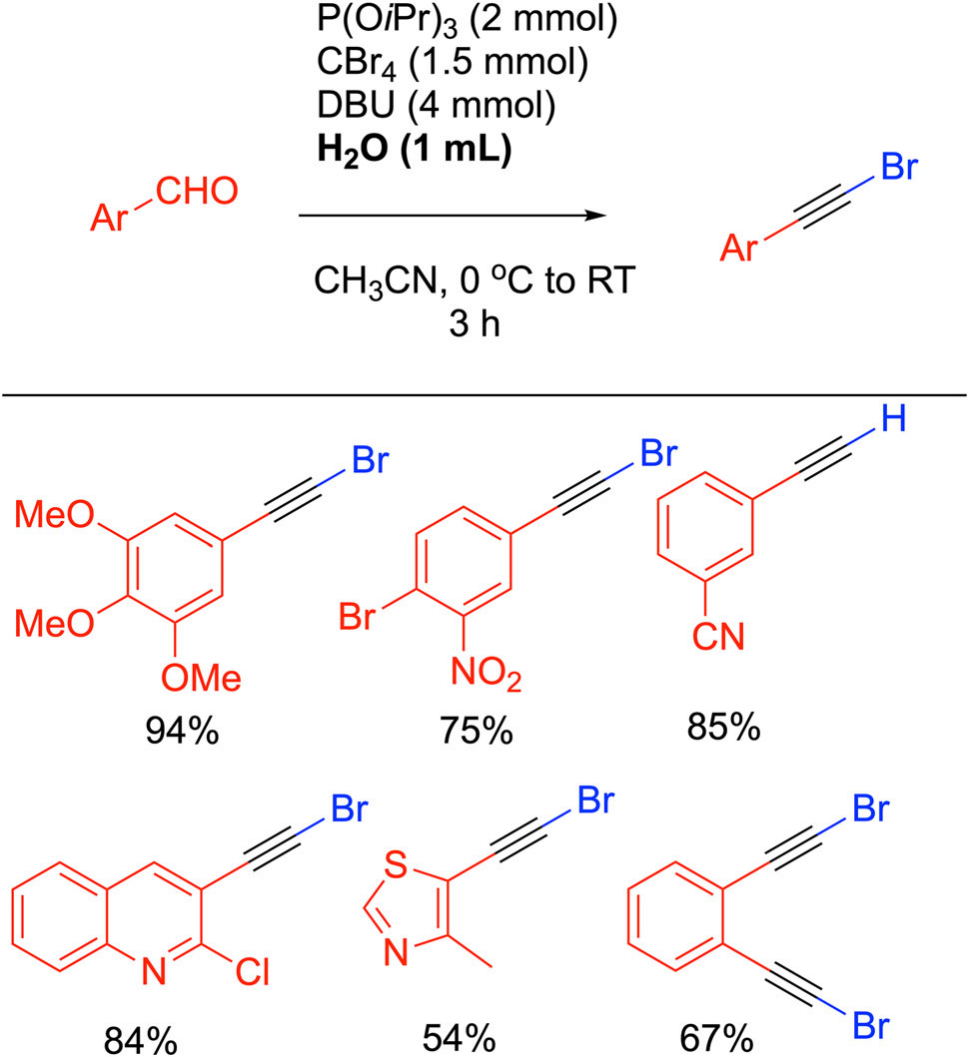
Doddi synthesis of 1‐bromo alkynes.

Like 1‐iodoalkynes, 1‐bromo alkynes are valuable coupling partners in transition‐metal‐catalyzed processes. As noted in Scheme 5, Doddi developed a modification of the Corey‐Fuchs reaction for the synthesis of terminal alkynes. The same authors found that a slight modification of the reaction conditions, which involved substitution of water for NaOH, led to the isolation of the intermediate 1‐bromoalkyne in high yields (Scheme 33). The difference in reaction outcomes between the formation of 1‐bromoalkynes (Scheme 33) versus terminal alkynes (Scheme 5) was explained by water forming a hydrogen bond with the DBU base, inhibiting its nucleophilic attack on the C─Br bond of the generated 1‐bromoalkynes and shutting down the debromination to the carbanion, ^−^C≡C─Ar.^[^ ^31^ ^]^

### 4.10 Diborylalkane

**Scheme 34:**
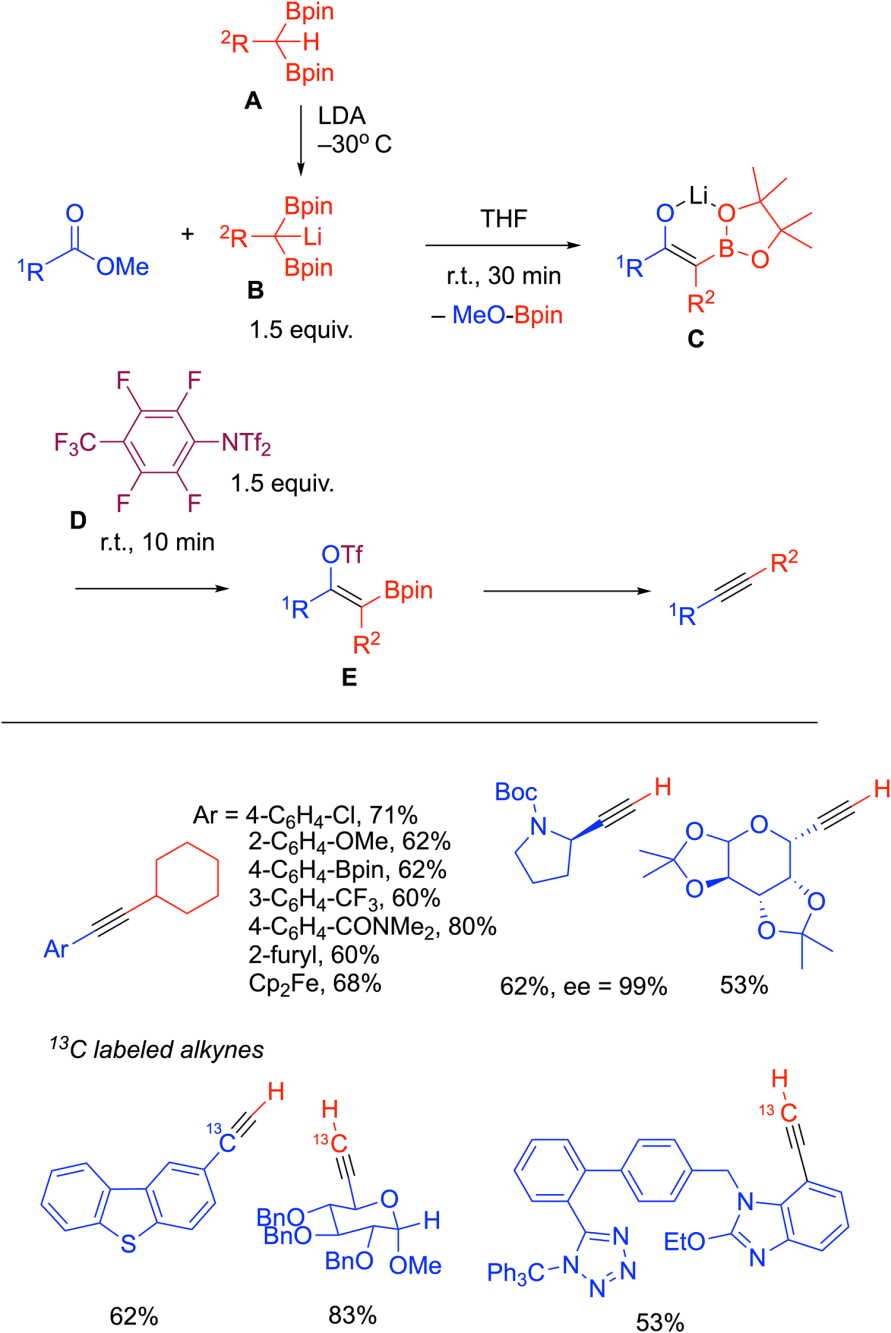
Internal and terminal alkynes from diborylalkynes.

The Liu group developed a method for the synthesis of internal and terminal alkynes in a one‐pot manner using diborylalkanes (Scheme 34).^[^ ^114^ ^]^ Esters are used in this procedure as the electrophile, which are more stable than aldehyde‐based precursors often used in alkyne syntheses. The procedure begins with deprotonation of gem‐diborylalkanes (**A**) with LDA at −30 °C to generate key 1,1‐diborylalkyl)lithiums (**B**) (formed in situ or isolated and stored in a dry box). Subjecting the ester to the 1,1‐(diborylalkyl)lithium (**B**) for 30 minutes at rt provides α‐B(pin) enolate derivatives (**C**), which were characterized by X‐ray crystallography. To activate the enolate, a fluorinated aryl triflimide (**D**) was used to form the alkenyl triflate (**E**). Finally, elimination of (Pin)BOTf from **E** leads to the formation of the alkyne. This method is mild and tolerates a variety of functional groups on the methyl benzoate partner. An array of functionalized alkyl groups were employed on the 1,1‐(diborylalkyl)lithium [**B**, R─C(BPin)_2_Li], enabling the application of this method to a multitude of alkynes (over 40 examples, including use of enantioenriched esters with α‐stereocenters). The reaction could also be scaled to produce gram quantities of alkynes.

This team also investigated the synthesis of ^13^C‐labeled alkynes (Scheme 34, bottom) using ^13^CH_2_(BPin)_2_, R^13^CO_2_Me, or both for double‐labeled alkynes (17 examples, 53–98% yields). Labeled terminal and internal alkynes were accessible using this approach. The labeling also facilitated mechanistic investigations.

This synthesis is promising because the procedure can be used to prepare a wide range of alkynes in short reaction times. The drawbacks include the need to synthesize the gem‐diborylalkanes, the formation of the 1,1‐diborylalkyl)lithiums at low temperature, and the synthesis of the triflating reagent. Given these constraints, it is anticipated that the value of this procedure will be in the synthesis of highly desirable labeled alkynes.

## 5 Outlook and Conclusions

The triple bonds of alkynes have very high energy content, making them reactive species that are useful in a wide range of C─C and C─heteroatom bond‐forming reactions. Alkynes are found in natural products,^[^ ^11^ ^]^ and are being increasingly employed in medicinal chemistry, including the Bergman cyclization^[^ ^115^, ^116^ ^]^ of endynes, which were found in powerful anti‐tumor agents like the calicheamicins.^[^ ^117^ ^]^ Alkynes have been applied extensively in the syntheses of heterocycles and used in azide‐alkyne click chemistry.^[^ ^118^, ^119^ ^]^ They are linchpins that span bioorganic chemistry, materials science, and supramolecular chemistry. Furthermore, researchers are taking advantage of alkynes’ bio‐orthogonality, and they are being used as imaging probes.^[^ ^120^ ^]^

The reader will notice that one of the challenges in the synthesis of alkynes outlined here and elsewhere stems from the same characteristic that makes them invaluable reactants: the high energy content of the C≡C bond. To make these energetic materials, high‐energy reagents and/or strong bases are par for the course. Herein, we have reviewed the historically most popular approaches to prepare all three bonds of alkynes' tripple bond that can be performed in a single reaction vessel without isolation of intermediates and under transition‐metal‐free conditions. Newer reactions and approaches are also outlined, with analysis of the strengths and weaknesses of each method. A goal of this review was to highlight new methods that are potentially more efficient and sustainable than their predecessors, which sometimes represent the first‐choice method because of their historical significance rather than being the most efficient and ecologically sound option.

## Supporting Information

The Supporting Information outlines the literature search parameters and full results that were used to prepare the graphs in Figures 2, 3, 4.

## Conflict of Interest

The authors declare no conflicts of interest.

## Supporting information



Supporting Information

## Data Availability

The data that support the findings of this study are available in the supplementary material of this article.
