# Supplementary material for: One‐Pot Transition‐Metal‐Free Methods for the Synthesis of All Three Bonds of the Alkyne's Triple Bond
Source: Chemistry. 2026 Jan 10;32(10):e03062. doi: 10.1002/chem.202503062 (PMC12995854; doi:10.1002/chem.202503062)
Supplement: Supplementary file 1 — Supporting Information [file CHEM-32-e03062-s001.pdf]

Supporting Information

One-Pot Transition Metal-Free Methods for the Synthesis of all Three Bonds of Alkynes

Eugene Kim,[a],‡ Yu Wu,[a],‡ Judith N. Carrano,[a] Jianyou Mao,[b],* Patrick J. Walsh[a],*

Dedicated to Madeleine Joullie, a force of change and a pioneering woman in Penn Chemistry.

‡These authors made equal contributions.

[a] E. Kim, Dr. Y. Wu, J. N. Carrano, Prof. P. J. Walsh
Department of Chemistry
University of Pennsylvania
213 S. 34th St. Philadelphia, PA 10104
E-mail: pwalsh@sas.upenn.edu

[b]. Prof. J. Mao
School of Chemistry and Molecular Engineering
Nanjing Tech University
30 South Puzhu Road, Nanjing 211816, P. R. C.

Table of Contents.

Literature Searches for Alkyne Substances..S2

Terminal Alkyne Substances Appearing in References....................................S2

Internal Alkyne Substances Appearing in References......................................S2

Comprehensive Set of Alkynes Appearing in Refs. and Literature Trends..........S3

Literature Searches for Alkyne Preparations..S8

Searches for Reactions that Prepare Terminal Alkynes......................................S8

Searches for Reactions That Prepare Internal Alkynes......................................S12

Searches for Sonogashira Couplings..S14

Literature Searches for Alkyne Substances

Searches were run in the CAS SciFinder^{n} database on May 21, 2025. The purpose was to obtain a comprehensive set of substances containing any alkynes and examine the characteristics of the answer set.

Terminal Alkyne Substances Appearing in References

The goal of this search was to retrieve a set of substances containing terminal alkynes. Substances should be organic (not containing metals), non-isotopic, and should not appear in multi-component systems. Each retrieved substance should appear in at least one reference indexed by CAS. In the Substances search context, we input the following substructure:

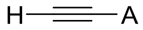

where A is any atom except H. Once the search was complete, we selected the substructure hits tab and filtered the results using the following options.

- **Number of Components:** 1
- **Isotopes:** Not containing isotopes
- **Metals:** Not containing metals
- **Reference Availability:** References available

This resulted in 1,400,411 substances, which were saved to the server for future use. It should be noted that, due to CAS indexing conventions, there is no easy way to exclude charged substances and radicals, so, those substances remain in the answer set. We will name this answer set ***SF-Subst-RCCH***.

Internal Alkyne Substances Appearing in References

The goal of this search was to retrieve a set of substances containing internal alkynes. Again, we sought substances that were organic (no metals), non-isotopic, single-component systems, and appearing in at least one reference indexed by CAS. In the Substances search context, we input the following substructure:

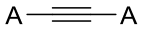

where A is any atom except H. Once the search was complete, we selected the substructure hits tab and filtered the results using the following options.

- **Number of Components:** 1
- **Isotopes:** Not containing isotopes
- **Metals:** Not containing metals
- **Reference Availability:** References available

This resulted in1,985,665 substances. Once again, it was not possible to remove charged or radical species. We will name this answer set ***SF-Subst-RCCR***.

Comprehensive Set of Alkynes Appearing in References and Literature Trends

In order to glean some publication trends about any type of alkyne published in a reference, we took the live answer set ***SF-Subst-RCCR*** and, using SciFinder^{n}'s Combine Searches feature, we merged that set with the previously-saved set ***SF-Subst-RCCH***. This resulted in 3,351,446 substances containing C≡C. We will name this answer set ***SF-Subst-Alkyne***.

From set ***SF-Subst-Alkyne***, we excluded the following:

- **Reaction Role:** Product
- **Reference Role:** Preparation; Synthetic preparation

We then filtered by the following criteria:

- **Commercial Availability:** Not available

566,983 substances are not commercially available, do not appear as the product of a reaction in the CASREACT database, and are not indexed as having been prepared in a reference appearing in CAPlus.

We next cleared all filters and exclusions to return to the full set ***SF-Subst-Alkyne***. We clicked **References** to retrieve all references including one or more of the substances in ***SF-Subst-Alkyne***. This resulted in 543,198 results. We will name this set ***SF-SubstRefs-Alkyne***.

We filtered these results by document type to see which types of references were being published and in which year.

- Filtering ***SF- SubstRefs-Alkyne*** by Document Type = Patent yielded 140,031 results. We filtered these references by publication year and exported the results.
- Filtering ***SF- SubstRefs-Alkyne*** by Document Type = Journal; Preprint yielded 395,297 results. We filtered these references by publication year and exported the results.
- Excluding the Document Type = Journal; Preprint; Patent from ***SF- SubstRefs-Alkyne*** yielded 7,996 results. (Resulting document types were review, book, conference, dissertation, report. Note that review articles appearing in journals are tagged with two document types, Journal and Review, so, none of the resulting reviews appeared in journals.) We filtered these references by publication year and exported the results.

We combined the three sets of data to yield the following table:

Publication Year	Journal Articles and Preprints	Patents	Other Document Types
**1831**	1	0	0
**1834**	1	0	0
**1835**	1	0	0
**1844**	1	0	0
**1845**	2	0	0
**1846**	2	0	0
**1851**	1	0	0
**1853**	2	0	0
**1854**	1	0	0

**1855**	1	0	0
**1857**	1	0	0
**1858**	4	0	0
**1860**	2	0	0
**1861**	2	0	0
**1862**	2	0	0
**1863**	1	0	0
**1864**	4	0	0
**1865**	10	0	0
**1866**	12	0	0
**1867**	5	0	0
**1868**	2	0	0
**1869**	7	0	0
**1870**	13	0	0
**1871**	10	0	0
**1872**	14	0	0
**1873**	13	0	0
**1874**	6	0	0
**1875**	12	0	0
**1876**	9	0	0
**1877**	12	0	0
**1878**	14	0	0
**1879**	9	0	0
**1880**	13	7	0
**1881**	24	2	0
**1882**	21	0	0
**1883**	8	0	0
**1884**	12	0	0
**1885**	5	0	0
**1886**	6	0	0
**1887**	18	0	0
**1888**	16	0	0
**1889**	15	0	0
**1890**	20	0	0
**1891**	13	1	0
**1892**	33	0	0
**1893**	22	0	0
**1894**	20	0	0
**1895**	18	0	0
**1896**	11	0	0
**1897**	14	1	0

**1898**	15	0	0
**1899**	29	0	0
**1900**	22	0	0
**1901**	23	1	3
**1902**	25	2	0
**1903**	29	1	0
**1904**	29	0	0
**1905**	35	1	0
**1906**	25	2	0
**1907**	35	1	0
**1908**	35	1	0
**1909**	39	1	0
**1910**	47	0	0
**1911**	51	1	0
**1912**	40	4	0
**1913**	55	3	0
**1914**	50	4	0
**1915**	19	2	0
**1916**	21	7	0
**1917**	13	2	0
**1918**	28	1	0
**1919**	20	0	0
**1920**	46	2	0
**1921**	47	1	0
**1922**	61	3	0
**1923**	69	0	0
**1924**	64	0	0
**1925**	82	3	0
**1926**	97	5	0
**1927**	88	4	0
**1928**	73	10	0
**1929**	94	4	0
**1930**	89	9	0
**1931**	77	14	0
**1932**	87	22	0
**1933**	176	36	0
**1934**	117	43	0
**1935**	177	20	0
**1936**	160	58	0
**1937**	177	41	0
**1938**	183	71	0

**1939**	187	81	0
**1940**	188	61	0
**1941**	157	65	0
**1942**	151	40	0
**1943**	109	41	0
**1944**	77	25	0
**1945**	120	50	0
**1946**	170	54	0
**1947**	201	50	0
**1948**	244	60	0
**1949**	310	123	1
**1950**	376	59	2
**1951**	341	96	2
**1952**	425	129	0
**1953**	514	147	0
**1954**	535	132	7
**1955**	645	179	0
**1956**	840	176	3
**1957**	855	275	8
**1958**	830	282	5
**1959**	985	299	14
**1960**	1088	335	5
**1961**	1214	310	10
**1962**	1330	483	14
**1963**	1726	460	16
**1964**	2099	542	37
**1965**	2338	645	29
**1966**	2391	672	49
**1967**	2213	515	52
**1968**	2384	506	83
**1969**	2232	513	71
**1970**	1723	375	68
**1971**	1842	372	72
**1972**	2104	503	260
**1973**	2247	591	98
**1974**	2400	586	130
**1975**	2424	683	279
**1976**	2383	696	111
**1977**	2539	656	107
**1978**	2476	558	90
**1979**	2658	563	122

**1980**	2748	675	124
**1981**	2734	647	108
**1982**	2872	625	91
**1983**	3078	677	114
**1984**	2998	719	123
**1985**	3379	785	104
**1986**	3506	863	68
**1987**	3747	908	66
**1988**	3846	933	47
**1989**	4010	874	51
**1990**	4102	918	68
**1991**	4117	947	70
**1992**	4195	1085	78
**1993**	4467	995	85
**1994**	4451	1037	78
**1995**	4633	1121	63
**1996**	4683	1172	89
**1997**	4812	1144	104
**1998**	5121	1422	70
**1999**	5229	1328	93
**2000**	5449	1550	90
**2001**	5701	1716	1663
**2002**	5977	2042	123
**2003**	6162	2222	147
**2004**	6544	2441	191
**2005**	7076	2542	192
**2006**	7642	2608	206
**2007**	8040	2809	502
**2008**	8392	3105	250
**2009**	8986	3342	218
**2010**	9704	3420	162
**2011**	10810	3478	153
**2012**	11744	4197	135
**2013**	12493	4574	130
**2014**	12668	5228	124
**2015**	12849	5227	86
**2016**	12695	5050	74
**2017**	12530	5164	28
**2018**	12410	5385	15
**2019**	12942	5985	19
**2020**	12915	5781	29

**2021**	13677	6276	6
**2022**	13605	6639	5
**2023**	13679	7123	2
**2024**	14010	7596	0
**2025**	4046	2257	0

Returning to the full set ***SF-SubstRefs-Alkyne***, we exported the concept filter data to see which roles indexers had assigned to the alkyne-containing substances in the references. The top 20 roles appear in the text of the article.

Literature Searches for Alkyne Preparations

Searches were run in the CAS SciFinder^{n} database on May 20, 2025, and May 29, 2025, with the purpose of finding all reactions that synthesize alkynes in a single step through the formation of all three bonds between the two carbons. In other words, the carbons on either side of the C≡C of the product should not be connected to one another in the reactant. It is important to note that Chemical Abstracts Service indexes reactions with both steps and stages. A *step* is a set of reaction conditions that results in an isolated intermediate. A *stage* is a set of reaction conditions, after which no intermediate is isolated. A reaction may have multiple stages for any given step. Although we were able to restrict our results to single *step* reactions, we were not able to restrict the number of stages. In addition, when filtering by reagent, visual inspection was the only way to ascertain which reagents had been used together in a stage and which reagents had been used in separate stages of the reaction.

Searches for Reactions that Prepare Terminal Alkynes

The initial set of queries was run on May 20, 2025, and updated on May 29, 2025, as additional reactions had been published in the nine intervening days.

We ran the following query in the Reactions search context in SciFinder:

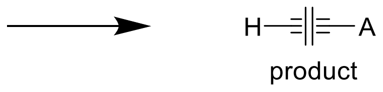

A is any atom except H. The alkyne substance is marked as a product, and the bond between the two carbons is tagged using the “make/break bond” tool to indicate that it should be formed in the course of the reaction. We clicked the tab to look at the substructure results.

From these substructure results, we excluded reactions that had any kind of bond between the two carbons in the reactant, employing the following structure:

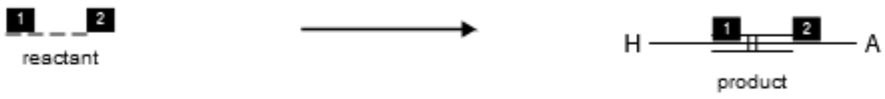

Here, we mark the bond between the two carbons in the reactant as an "unspecified bond," meaning that it can be single, double, or triple. We map atoms to show that the carbons are conserved from reactant to product.

From the resulting set, we perform another structure exclusion, this time for any products containing a metal atom, as in the following scheme:

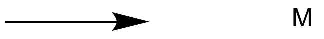

product

These results seemed to form the alkyne. We then filtered the set in the following ways:

- **Number of Steps:** 1
- **Reaction Mapping:** Mapping data available

From these results, we excluded any that used the following 58 catalysts that contain transition metals, lanthanides, and actinides:

- Tetrakis(triphenylphosphine)palladium
- Chloro(η^5-cyclopentadienyl)bis(triphenylphosphine)ruthenium
- Cuprous iodide
- Palladium
- Grubbs second generation catalyst
- Osmate (OsO_4^{1-}), potassium, (*T*-4)-
- Dichlorobis(triphenylphosphine)palladium
- Cuprous chloride
- Osmium tetroxide
- Palladium chloride
- Palladium dihydroxide
- Palladium diacetate
- Potassium osmate dihydrate
- Tris(dibenzylideneacetone)dipalladium
- Scandium triflate
- [1,1'-Bis(diphenylphosphino)ferrocene]dichloropalladium
- Bis(benzonitrile)dichloropalladium
- Cesium carbonate
- Copper sulfate
- Cupric chloride
- Manganese oxide (MnO_2)
- Ruthenate (RuO_4^{2-}), sodium (1:2), (*T*-4)-
- Ruthenium(1+), (acetonitrile)(η^5-2,4-cyclopentadien-1-yl)bis[2-(diphenylphosphino-κ*P*)-6-(5'-phenyl[1,1':3',1''-terphenyl]-2'-yl)pyridine]-, hexafluorophosphate(1-) (1:1)
- Silver nitrite
- Zinc
- Chloro[(1,2,3,4,5,6-η)-1-methyl-4-(1-methylethyl)benzene][2-(1*H*-pyrazol-1-yl-κN^2)phenyl-κ*C*]ruthenium
- Stereoisomer of di-μ-chlorotetrakis[2-(1*H*-pyrazol-1-yl-κN^2)phenyl-κ*C*]dirhodium
- Stereoisomer of di-μ-chlorotetrakis[2-(1*H*-pyrazol-1-yl-κN^2)phenyl-κ*C*]diiridium

- Cobalt
- Copper
- Dichloro(η^6-*p*-Cymene)(triphenylphosphine)ruthenium
- 1,1-Bis(diphenylphosphino)ferrocene
- (1*R*)-1-(Diphenylphosphino)-2-[(1*R*)-1-[(2-pyridinylmethylene)amino]ethyl]ferrocene
- Bis[μ-[2-[(4*S*,5*S*)-4,5-diphenyl-3-[2,4,6-tris(1-methylethyl)phenyl]-1-imidazolidinyl-κ*C*^{2}]benzenesulfonato(3-)-κ*O*]]disilver
- Cerium
- Cobalt chloride ($CoCl_2$)
- Copper(1+), tetrakis(acetonitrile)-, (*T*-4)-, perchlorate (1:1)
- Copper acetylide ($Cu(C_2H)$)
- Copper bromide (CuBr)
- Dibromo[1,1'-(oxy-κ*O*)bis[2-(methoxy-κ*O*)ethane]]nickel
- Dicobalt octacarbonyl
- Gold trichloride
- Indium trichloride
- Magnesium
- Manganese
- Molybdenum
- Nickel
- Nickel dichloride
- Palladium, tris[μ-[(1,2-η:4,5-η)-(1*E*,4*E*)-1,5-diphenyl-1,4-pentadien-3-one]]di-, compd. with trichloromethane (1:1)
- Platinum dioxide
- Ruthenium
- Silver
- (*SP*-4-1)-Dichlorobis(triphenylphosphine)palladium
- (*SP*-4-2)-Chlorotris(triphenylphosphine)rhodium
- stereoisomer of Chloro[4-methyl-*N*-[(1*S*,2*S*)-2-[(*R*)-[2-[[(1,2,3,4,5,6-η)-4-methylphenyl]methoxy]ethyl]amino-κ*N*]-1,2-diphenylethyl]benzenesulfonamidato-κ*N*]ruthenium
- Titania
- Zinc oxide (ZnO)
- Grubbs' catalyst

This resulted in 6855 reactions. We will name this set ***SF-Rxn-RCCH***. We filtered the set to determine the relative yields; 1997 of the reactions had no yield indexed. The yields of the remaining reactions are as follows:

Yield	Number of Reactions	Percentage of Reactions
90-100%	851	17.5%
80-89%	1045	21.5%
70-79%	957	19.7%
50-69%	1229	25.3%

30-49%	566	11.7%
10-29%	195	4.0%
<10%	15	0.3%

Clicking on the "References" link from ***SF-Rxn-RCCH*** led to a set of 3170 references, published between 1955-2025.

Returning to the reaction set, a manual screen of the first few pages of reactions from ***SF-Rxn-RCCH*** indicated that many employed reactants with a common substructure. To explore the reactions that included such a substance, we searched within the set for the following reaction:

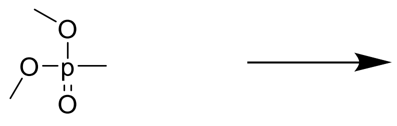

We searched for this as a substructure and retrieved 5312 reactions. Browsing the reactions retrieved, it seems that many convert an aldehyde to an alkyne. To examine the reactions that perform that transformation, we searched within the 5312 results for the following reaction substructure:

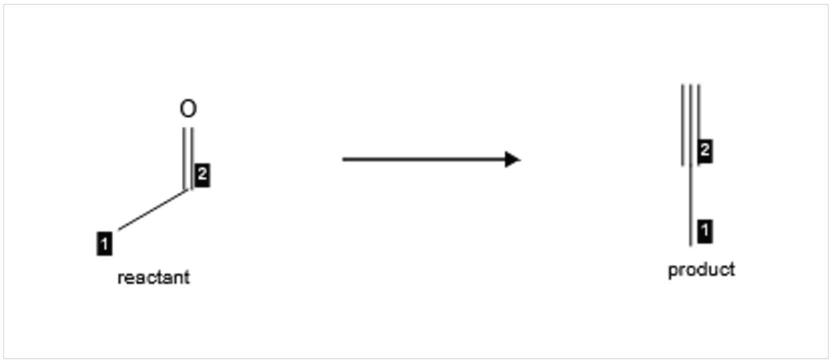

This search resulted in 4809 results. Filtering by reagent indicated that the majority of these reactions employ potassium carbonate (3932) or cesium carbonate (432) in one or more stages of the reaction. Limiting to these two reagents and filtering by solvent indicates that most of these reactions are done in methanol and that their yields are as follows:

Yield	Number of Reactions
90-100%	688
80-89%	742
70-79%	620
50-69%	773
30-49%	355
10-29%	130
<10%	10

Yield Not Indexed	1,491

To attempt to extract trends about the reactions that do not employ the phorphorus-containing reactants, we returned to ***SF-Rxn-RCCH*** and excluded the following reaction structure:

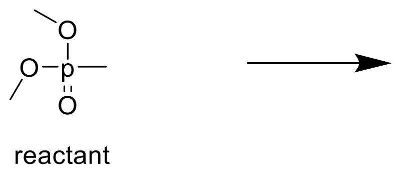

This yielded 1543 results. Filtering by reagent, many of the reactions employ butyllithium (615) and/or triphenylphosphine (435). Once again, it was difficult to determine which reagents were used together in the same stage of the reaction and which reagents were used in separate stages.

Attempts to group reactions by transformation led to confusing results; therefore, this strategy was not employed.

Searches for Reactions That Prepare Internal Alkynes

The initial set of queries was run on May 20, 2025. The results for these queries were used, as queries run on May 29, 2025, yielded no additional hits.

We initiated the following search in the Reactions context of SciFinder^n:

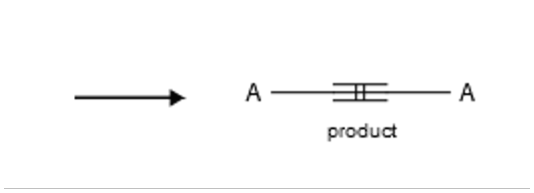

where A is any atom but H. After clicking the tab to the substructure results, the following filters were applied to the results:

- **Number of Steps:** 1
- **Reaction Mapping:** Mapping data available

We excluded the following substructure reaction scheme from our search results:

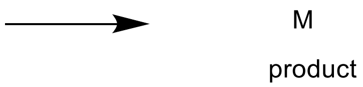

This ensured that the product contained no metals. We then excluded this reaction substructure from the ensuing result set:

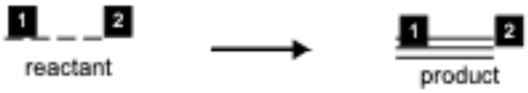

The result was a set of substances that formed a triple bond from two unbonded carbon atoms. We saved this query to the server for future use, and we will refer to it as ***SF-Rxn-RCCR***.

We tried a different approach to removing those reactions catalyzed by transition metals. From the reaction set ***SF-Rxn-RCCR***, we clicked on the References button to collect the 976 articles that described them. From there, we clicked on the Substances button to retrieve the 80,015 substances indexed in those references. We applied the following filters to the substance set:

- **Reaction Role:** Catalyst
- **Element:** Choose all transition metal elements

From this set of substances, we clicked the Reactions button and filtered by Reaction Role = Catalyst.

We then used the Combine Search Sets feature to subtract this live set from the set ***SF-Rxn-RCCR***, and we excluded by catalyst to remove any additional catalysts including transition metals or the elements U, Yb, Eu, La, and Ce. To ensure that the retrieved reactions do not contain an alkyne starting material, we excluded the following substructure:

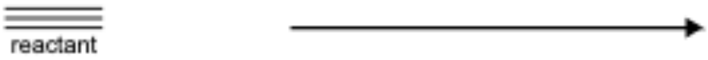

This resulted in a set of 2067 reactions. We filtered the set by yield to determine the range of yields observed; 567 of the reactions did not have indexed yields. The following chart shows the total number of reactions with yields indexed in SciFinder, arranged by yield bracket.

Yield	Number of Reactions	Percentage of Reactions
90-100%	250	16.7%
80-89%	340	22.7%
70-79%	297	19.8%
50-69%	376	25.1%
30-49%	171	11.4%
10-29%	58	3.9%
<10%	8	0.5%

Filtering by reagent indicated the following trends:

- 30% employ triphenylphosphine (618)

- 28% employ butyllithium (586)
- 21.7% use water (449)
- 21.5% use lithium bis(trimethylsilyl)amide (445)
- 21.5 use ammonium chloride (442)

It is difficult to tell which reagents are used together or to extrapolate from reagents the potential named reactions being used for this purpose, but saving and intersecting the sets with the most commonly used reagents (triphenylphosphine and butyllithium) indicate that 376 reactions use these two reagents, either in the same stage or in different stages, to form the alkyne.

Searches for Sonogashira Couplings

For comparison purposes, we also attempted a search in SciFinder for Sonogashira couplings. These searches were run in June 2025. We determined that the easiest way to ensure that a transformation was a Sonogashira coupling was to control the catalyst. Therefore, we began with the following search for palladium-catalyzed reactions.

In the SciFinder^{n} substance context, we searched for all substances with the following substructure:

Pd

We filtered to those that had the reaction role of catalyst and linked to related reactions. From here, we searched within our results for the following substructure:

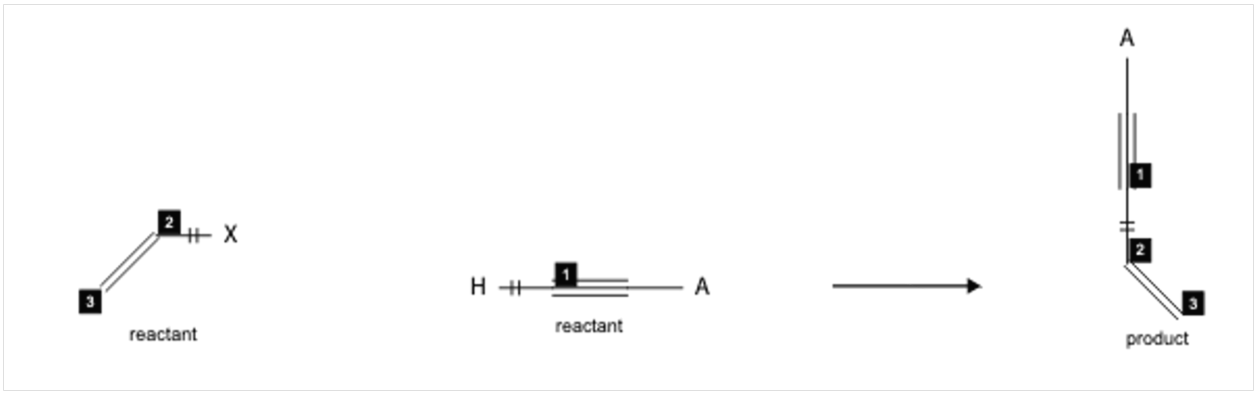

We used bond marking in the reactant substructures to ensure that the C-X and C-H bonds were broken, and we used atom mapping from reactant to product, combined with bond marking in the product substructure, to force the system to retrieve reactions that formed the desired C-C bond. We chose the substructure tab of results and applied the following filters:

- **Number of Steps:** 1
- **Catalyst:** Cuprous iodide

This resulted in 16,870 reactions. We saved our query to the server; we will call it ***SF-Rxn-SC_CuICat***. We then removed the catalyst filter and, instead applied the following filter:

- **Reagent:** Cuprous iodide

This resulted in 6326 results. We used the Combine Search Sets feature to merge this live set with the saved ***SF-Rxn-SC_CuICat*** results to obtain a total of 174,174 reactions that employ both a palladium catalyst and cuprous iodide. We filtered the merged set by yield to determine the range of yields observed; 60,651 of the reactions did not have indexed yields. The following chart shows the total number of reactions with yields indexed in SciFinder, arranged by yield bracket.

Yield	Number of Reactions	Percentage of Reactions
90-100%	23,451	20.7%
80-89%	23,871	21.0%
70-79%	19,956	17.6%
50-69%	24,868	21.9%
30-49%	13,602	12.0%
10-29%	6,762	6.0%
<10%	1,014	0.9%

An examination of the publication years led to the following information:

Publication Year	Reactions Forming RCCH without Metals	Reactions Forming RCCR without Metals	Sonogashira-Type Couplings
1939	0	1	0
1940	0	0	0
1941	0	0	0
1942	0	0	0
1943	0	0	0
1944	0	0	0
1945	0	0	0
1946	0	0	0
1947	0	0	0
1948	0	0	0
1949	0	0	0
1950	0	0	0
1951	0	0	0
1952	0	0	0
1953	0	0	0
1954	0	0	0
1955	1	1	0
1956	0	0	0
1957	0	0	0
1958	0	0	0

1959	1	0	0
1960	0	0	0
1961	0	1	0
1962	0	0	0
1963	0	0	0
1964	0	1	0
1965	0	1	0
1966	0	0	0
1967	0	1	0
1968	0	1	0
1969	0	2	0
1970	0	0	0
1971	0	0	0
1972	1	0	0
1973	0	1	0
1974	1	3	0
1975	0	0	0
1976	0	0	0
1977	14	7	0
1978	2	2	1
1979	1	4	0
1980	15	2	15
1981	0	5	2
1982	9	4	8
1983	4	4	4
1984	4	11	6
1985	5	5	83
1986	8	12	83
1987	14	14	83
1988	6	8	137
1989	11	10	80
1990	6	11	89
1991	5	4	33
1992	6	9	70
1993	5	8	107
1994	8	2	132
1995	12	4	240

1996	22	10	124
1997	12	11	242
1998	17	134	518
1999	25	117	928
2000	60	9	1439
2001	65	12	2191
2002	66	10	1976
2003	65	39	2585
2004	149	63	3038
2005	118	16	3845
2006	138	127	3774
2007	205	83	4217
2008	292	86	5204
2009	262	41	5953
2010	220	27	6327
2011	202	40	7138
2012	270	48	7644
2013	307	39	8002
2014	426	28	8802
2015	471	99	9101
2016	304	45	8478
2017	295	76	8337
2018	218	34	9406
2019	363	96	8601
2020	315	31	9568
2021	386	171	10724
2022	433	86	10562
2023	450	53	11415
2024	421	226	9952
2025	139	71	2910
